# Spark-like T cell represents a novel functional state of CD8^+^T cells associated with anti-PD-1 immunotherapy response and clinical prognosis in non-small cell lung cancer

**DOI:** 10.3389/fimmu.2026.1997857

**Published:** 2026-10-28

**Authors:** Zhuoqi Li, Rui Li, Feng Sun, Chunyan Han, Mei Tian, Yinghua Song, Wenhong Xu, Lu Zhang, Fujing Huang, Xiao Wen, Xiaoshu Yin, Xiaoyan Lin, Yuan Tian

**Affiliations:** 1 Department of Radiotherapy Oncology, Affiliated Hospital of Shandong University of Traditional Chinese Medicine, Jinan, China; 2 Department of Pathology, Shandong Provincial Hospital Affiliated to Shandong First Medical University, Jinan, China; 3 Department of Respiratory and Critical Care Medicine, Affiliated Hospital of Shandong University of Traditional Chinese Medicine, Jinan, China; 4 Department of Respiratory and Critical Care Medicine, Shandong Second Provincial General Hospital (Shandong Provincial ENT Hospital), Jinan, China

**Keywords:** anti-PD-1 immunotherapy, CD8^+^T cells, multiplex immunohistochemistry, NSCLC, prognostic signature, single-cell RNA sequencing, Spark-like T cells

## Abstract

**Background:**

Spark-like T cells constitute a novel functional state of CD8^+^T cells and have emerged as key drivers of immunotherapy response in preclinical models, yet their clinical relevance in non-small cell lung cancer (NSCLC) remains undefined.

**Methods:**

Single-cell RNA sequencing (GSE207422), spatial transcriptomics (GSE271689), bulk RNA expression cohorts (TCGA-NSCLC, GSE37745, GSE41271, and GSE72094), and multiplex immunohistochemistry (mIHC) of a 100-patient cohort treated with anti-PD-1 were integrated to characterize Spark-like T cells and assess their clinical relevance. A 14-gene prognostic signature was constructed via LASSO-Cox regression.

**Results:**

Spark-like T cells were significantly enriched in anti-PD-1 responders in the single-cell RNA sequencing cohort. mIHC validation in the 100-patient cohort confirmed that high Spark-like T cell intensity in tumors was independently associated with prolonged overall survival (log-rank test, p < 0.001) and partial response to anti-PD-1 therapy (p = 0.0075). Spatial transcriptomics indicated strong co-localization of Spark-like T cells with M1 macrophages (Spearman r = 0.524, p = 3.4 × 10^-42^), suggesting their spatial co-enrichment with pro-inflammatory myeloid cells. In silico knockout identified TNF, ENTPD1 (CD39), and IL2 as cooperative regulators of Spark-like T cell effector function. The 14-gene Spark-like T cell-related signature (VDAC1, ITGB1, GALNT2, SEMA7A, UBB, SLC7A5, FURIN, SNX9, PSMB2, CXCL13, TOX, PPM1M, CCND2, and CLNK) robustly predicted overall survival across all four independent cohorts and remained an independent prognostic factor after adjustment for age, sex, and tumor stage. High-risk patients exhibited an immune-excluded, oncogene-driven tumor microenvironment with reduced infiltration of CD8^+^T cells.

**Conclusions:**

Spark-like T cells are clinically relevant determinants of anti-PD-1 response in NSCLC. mIHC quantification and the 14-gene signature provide promising translational tools for patient stratification and risk assessment.

## 1 Introduction

Lung cancer remains the leading cause of cancer-related mortality worldwide, with non-small cell lung cancer (NSCLC) accounting for approximately 85% of all cases (1). The advent of immune checkpoint inhibitors (ICIs) targeting the programmed death-1 (PD-1)/programmed death-ligand 1 (PD-L1) axis has substantially improved clinical outcomes for patients with advanced NSCLC (2). Nevertheless, a considerable proportion of patients exhibit primary resistance or develop acquired resistance during treatment, and heterogeneity in therapeutic response between individuals remains a major obstacle to further improving patient survival (3). Conventional predictive biomarkers, including tumor mutational burden (TMB) and PD-L1 expression, as well as emerging biomarkers, such as mutation clonality, an inflamed microenvironment, and alterations in individual genes provide only limited patient stratification and do not fully capture the complex functional heterogeneity of tumor-infiltrating T cells that underlies differential responses to immunotherapy (4).

CD8^+^ cytotoxic T lymphocytes are the principal effectors of anti-tumor immunity, and their abundance, differentiation state, and functional capacity within the tumor microenvironment (TME) are closely linked to the efficacy of ICI therapy (5, 6). However, CD8^+^T cells encompass a heterogeneous spectrum of functional states ranging from naive and stem-like memory cells to effector, terminally differentiated, and exhausted subtypes (7–9). Accumulating evidence indicates that the balance between these states, particularly between functionally competent effector cells and terminally exhausted cells, critically determines whether an anti-tumor immune response can be reinvigorated by checkpoint blockade (7–9). However, the precise identity of the CD8^+^T cell subtypes that most effectively mediate ICI response in NSCLC, and the molecular programs governing their activity remain incompletely defined.

Recently, a landmark study reported an extremely rare yet functionally indispensable CD8^+^T cell subtype, termed “Spark T cells”, as a key driver of stochastic variability in immunotherapy outcomes in a genetically uniform mouse model (10). By integrating single-cell analyses with a stochastic model of anti-tumor immunity, that study showed that response heterogeneity to immune checkpoint blockade could be traced to a minute CD8^+^T cell population whose abundance and functional state directly correlated with the magnitude of the anti-tumor immune response. These cells were defined by a characteristic molecular program, namely high expression of effector and immunoregulatory molecules including ENTPD1 (CD39), PTPRC (CD45RO), and CD2, alongside low expression of CCR7 and IL7R, which distinguishes them from conventional memory, effector, and exhausted CD8^+^T cell populations. Importantly, this population was discovered in the setting of preclinical immune checkpoint blockade, where it was demonstrated to be functionally critical for the efficacy of PD-1-targeted immunotherapy. These findings raise the possibility that rare Spark T cells may similarly serve as critical determinants of ICB efficacy in human cancers. To date, however, the relevance of Spark T cells in human tumors remains largely unexplored: their precise molecular identity, developmental trajectory, intercellular communication networks, and spatial ecological niche in the NSCLC tumor microenvironment (TME) have not been systematically characterized, and whether Spark T cell-associated transcriptional signatures can serve as clinically useful biomarkers for predicting prognosis and immunotherapy response in patients with NSCLC remains unclear.

To address these gaps, we employed an integrated multi-omics strategy combining single-cell RNA sequencing (scRNA-seq), spatial transcriptomics, bulk RNA-seq, and multiplex immunohistochemistry (mIHC). We first constructed a single-cell atlas of NSCLC tumors from patients treated with anti-PD-1 immunotherapy and identified a Spark-like T cell population enriched in responders. We then validated its clinical relevance in an independent 100-patient cohort using mIHC staining for CD8A, CD39 (ENTPD1), and CD45RO (PTPRC). We further delineated the pseudotime differentiation trajectory, cell-cell interaction landscape, and key regulatory targets of Spark-like T cells, and employed in silico knockout simulations to assess the functional necessity of core genes. Spatial transcriptomics was used to map the tissue distribution and microenvironmental co-localization of Spark-like T cells. Finally, we constructed and validated a 14-gene Spark-like T cell-related prognostic signature across multiple independent NSCLC cohorts, and characterized its association with the tumor immune microenvironment. Collectively, our findings provide a comprehensive multi-dimensional characterization of Spark-like T cells in NSCLC and establish their potential as both biological mediators and predictive biomarkers of anti-PD-1 immunotherapy response.

## 2 Materials and methods

### 2.1 Data collection and sample annotation

Single-cell RNA-seq (scRNA-seq) transcriptome data were downloaded from the Gene Expression Omnibus (GEO, https://www.ncbi.nlm.nih.gov/geo/) database under accession GSE207422. This dataset contains 14 surgical samples from NSCLC patients treated with neoadjuvant anti-PD-1-based immunotherapy, with tumor tissues collected at surgery after treatment. Bulk RNA-seq transcriptome and clinical data from TCGA-LUAD and TCGA-LUSC, including STAR-FPKM expression matrices, survival data with OS.time/OS.event, and clinical phenotype files, were obtained from The Cancer Genome Atlas database (TCGA, https://portal.gdc.cancer.gov). Bulk RNA expression data from GSE37745, GSE41271, and GSE72094 were obtained from the GEO database. Spatial transcriptome data from GSE271689, generated using the GeoMx DSP platform and comprising pkc probe annotation files,.dcc.gz raw count files, and tissue ROI information, were also downloaded from the GEO database.

The sample annotation strategy was as follows: samples in GSE207422 were classified as responders, non-responders, or Not Evaluable (NE) according to pathological response criteria (MPR vs. NMPR) and RECIST standards. TCGA data from both the LUAD and LUSC cohorts were integrated as TCGA-NSCLC, and used to construct the construction of Spark-like T cell-related prognostic signature. Bulk RNA expression data from GSE37745, GSE41271, and GSE72094 were used to validate the prognostic signature.

### 2.2 Preprocessing, quality control, and batch correction of scRNA-seq data

All downstream analyses were performed using R (v4.3.0) and Seurat (v5). For the GSE207422 dataset, UMI expression matrices were read using the R function “data.table::fread”, and sample metadata was read using “readxl::read_excel”. Sample information was extracted from cell barcodes following the rule paste(x[1], x[2], sep = “_”), and Seurat objects were constructed after the expression matrices were matched to the metadata. Quality control thresholds were set as follows: nFeature_RNA > 500 and < 5000, nCount_RNA > 1000 and < 23000, and percent.mt < 18% (mitochondrial gene ratios were calculated using PercentageFeatureSet with pattern = “^MT-”). Data were normalized using SCTransform (vst.flavor = “v2”) with regression variables vars.to.regress = c(“percent.mt”, “percent.rb”). Harmony batch correction was applied across the first 30 principal components of the SCT assay (batch variable = Sample, assay.use = “SCT”). Dimensionality reduction was performed using principal component analysis (PCA) (dims = 1:30) combined with UMAP (n.neighbors = 30, min.dist = 0.3, reduction = “harmony”). Clustering was performed using FindNeighbors (reduction = “harmony”, dims = 1:30) with multi-resolution testing (resolutions = 0.2, 0.4, 0.6, 0.8, 1.0, 1.2), and resolution = 0.8 was ultimately selected for downstream cell type annotation.

### 2.3 Cell type annotation and CD8^+^T cell subtype identification

Cells were annotated based on canonical marker genes as follows: epithelial cells (EPCAM, KRT5, KRT19, SFN), CD8^+^T cells (CD8A, CD8B, CD3D, CD3E), CD4^+^T cells (CD4, IL7R), B cells (CD79A, MS4A1), myeloid cells (CD68, CD14, ITGAM, LYZ), mast cells (TPSAB1, CPA3, KIT), fibroblasts (COL1A1, COL1A2, PDGFRA, VIM), NK cells (NCAM1, NKG7, KLRD1, GNLY), and plasma cells (JCHAIN, MZB1, IGKC, IGHG1). The R function “AverageExpression” was used to compute average expression across cell types, and a dot product strategy was applied to select one representative marker gene per cell type. Annotation accuracy was verified using “FeaturePlot” and “DotPlot”.

CD8^+^T cells were extracted from the annotated object, and normalization using the R function “SCTransform” (regressing percent.mt and percent.rb) and “Harmony” batch correction (group.by.vars = “Sample”) were re-applied. PCA (dims = 1:30) and UMAP dimensionality reduction (reduction = “Harmony”) were performed, with clustering resolution set to 0.4. Four functional modules were constructed to access the core features of Spark-like T cells: Spark_Core (ENTPD1, CD2, CCL5, IFNG, TNF, IL2, GZMB, PRF1, EOMES, RUNX3), Cytotoxic (GZMA, GZMB, PRF1, GNLY, NKG7), Stem_Memory (TCF7, LEF1, CCR7, SELL, IL7R), and Exhaustion (PDCD1, HAVCR2, LAG3, TIGIT, CTLA4, TOX). Module scores were computed using AddModuleScore (assay = “SCT”, search = FALSE).

Spark-like T cells were identified using a literature-based gating approach (10): first, markers defined in the literature (CCR7, PTPRC, ENTPD1, CD2, IL7R) were scored by percentile ranking (0–1 normalization), with high scores assigned to low expression of CCR7 and IL7R and high expression of the remaining markers. The average of the five marker scores was used to derive a composite phenotype score (Spark_pheno_score). Clusters with phenotype scores in the top 10% and Spark_Core module scores at or above the median were defined as Spark-like T cells. The remaining clusters were annotated as Naive_TCM, TEM, TEMRA, Tex, and Other based on canonical marker specificity scores.

Differentially expressed genes between Spark-like T cells and other types of cells were identified using the R function “FindMarkers” (Wilcoxon rank-sum test, assay = “SCT”, slot = “data”, min.pct = 0.1, logfc.threshold = 0.25, only.pos = TRUE), followed by gene ontology biological process (GO-BP)/Kyoto Encyclopedia of Genes and Genomes (KEGG) enrichment analysis (clusterProfiler, OrgDb = org.Hs.eg.db, pAdjustMethod = “BH”, pvalueCutoff = 0.05). Hallmark (category = “H”) and C7 immune gene sets were obtained via the R package “msigdbr”, and pathway activities across samples were computed using gene set variation analysis (GSVA) (method = “ssgsea”, kcdf = “Gaussian”). Differences between responders and non-responders were assessed using the Wilcoxon rank-sum test.

### 2.4 Clinical sample collection and mIHC experiment

NSCLC specimens were collected from patients who received anti-PD-1 immunotherapy at the Department of Pathology, Shandong Provincial Hospital, between 2020 and 2026. In accordance with the treatment methods of patients in the scRNA-seq cohort GSE207422, all 100 NSCLC samples used for mIHC were primary tumor tissues obtained from surgical resection specimens after the treatment of anti-PD-1 immunotherapy combined with chemotherapy. Clinicopathological staging was determined according to the IASLC/UICC TNM classification, 8th edition. Written informed consent was obtained from all participants. Tumor tissues were obtained from excised biopsy specimens were formalin-fixed and paraffin-embedded (FFPE) for histological evaluation. The baseline clinical characteristics of the 100 patients with NSCLC included in this study are summarized in **Table 1**.

**Table 1:** The basic clinical information of the NSCLC patients enrolled in the IHC experiments.

Characteristics	Levels	Overall (%)
n		100
Age, n (%)	<60	35 (35)
	>=60	65 (65)
Gender, n (%)	Male	86 (86)
	Female	14 (14)
Histologic type, n (%)		
	Adenocarcinoma	27 (27)
	Squamous cell carcinoma	72 (72)
	Adenosquamous carcinoma	1 (1)
T stage, n (%)	T1	11 (11)
	T2	29 (29)
	T3	38 (38)
	T4	22 (22)
N stage, n (%)	N0	19 (19)
	N1	16 (16)
	N2	58 (58)
	N3	7 (7)
M stage, n (%)		
	M0	100 (100)
Clinical stage, n (%)		
	Stage I	23 (23)
	Stage II	18 (18)
	Stage III	59 (59)
Response, n (%)		
	SD	62 (62)
	PR	38 (38)
Survival status, n (%)		
	Alive	47 (47)
	Dead	53 (53)

All 100 samples were primary tumor tissues obtained from surgical resection specimens after the treatment of anti-PD-1 immunotherapy combined with chemotherapy. Clinicopathological staging was determined according to the IASLC/UICC TNM classification, 8th edition.

For multiplex immunohistochemistry (mIHC), FFPE samples were first cut into thin sections. The sections were then deparaffinized and subjected to antigen retrieval, blocking of endogenous peroxidase activity and non-specific binding. In each staining cycle, the sections were incubated overnight at 4 °C with a specific primary antibody, followed by incubation with an HRP-conjugated secondary antibody for 1 h at room temperature. Tyramide signal amplification (TSA) fluorophores (AiFang Biological, China; AFIHC024) were then applied for 8 min. The markers were selected on the basis of the scRNA-seq-defined Spark-like program: CD39 (ENTPD1) and CD45RO (PTPRC) are core genes of the Spark-like T cell signature, and CD8A was used as the CD8^+^T cell lineage gate. Antibodies were subsequently eluted by heating the sections in antigen retrieval buffer at boiling temperature for 20 min. After repeating this cycle, the sections were sequentially incubated with primary and secondary antibodies, followed by TSA fluorophores (TYR-520, TYR-570, and TYR-690) in the prescribed order. Finally, nuclei were counterstained with DAPI for 10 min, and the sections were scanned using a fluorescence panoramic slide scanning system (LYMPUS VS200 ASW 3.2.1). The primary antibodies used were as follows: anti-CD8A (KeyGEN BioTECH, China; KGC61433-1, 1:1000), anti-CD39 (KeyGEN BioTECH, China; KGC62277-2, 1:100), and anti-CD45RO (Proteintech, China; 65150-1-Ig, 1:1000). The specificity of the primary antibodies was verified on the basis of the manufacturers’ validation information, including the positive-control tissues recommended in the product datasheets and on the manufacturers’ official websites: mouse spleen tissue for CD8A (https://source.keygentec.com.cn/pdf/20260715103628933976.pdf), Raw264.7 cells for CD39 (https://source.keygentec.com.cn/pdf/20260715103628992095.pdf), and human colon cancer tissue for CD45RO (https://www.ptgcn.com/products/pictures/pdf/65150-1-Ig.pdf).

### 2.5 Spark-like T cell differentiation trajectory analysis

Dynamic trajectories of CD8^+^T cells were constructed using Monocle3. Valid functional states including Spark-like T, Naive_TCM, TEM, TEMRA, and Tex, were extracted, and SCT assay count matrices were used to construct a “CellDataSet” (new_cell_data_set). After “preprocess_cds” (num_dim = 30, method = “PCA”) and “align_cds” (alignment_group = “Sample”) batch correction, reduce_dimension (reduction_method = “UMAP”, preprocess_method = “PCA”, umap.min_dist = 0.3), cluster_cells (resolution = 0.4), and learn_graph were executed. Naive_TCM cells or cells with Stem_Memory module scores in the top 10% were designated as root nodes (root_cells), and “order_cells” was applied to compute pseudotime. The R function “graph_test” (neighbor_graph = “principal_graph”, cores = 1) was used to identify genes with significant expression changes along pseudotime. After q-value filtering, the top 50 genes were retained. Pseudotime was divided into 50 bins, and the average expression within each bin was computed to construct heatmaps comparing gene expression along the trajectory between responders and non-responders. Expression dynamics of specific genes (CCR7, PTPRC, ENTPD1, CD2, IL7R, etc.) along pseudotime were visualized using the function “plot_genes_in_pseudotime” (color_cells_by = “CD8_subtype”). Differences in the pseudotime distribution of Spark-like T cells between responders and non-responders with NSCLC were assessed using the Wilcoxon rank-sum test.

### 2.6 Cell-cell interaction network analysis across treatment response groups

Spark-like T cell populations were mapped back to the full GSE207422 dataset, and celltype_chat grouping was constructed (retaining Spark-like_T, Naive_TCM, TEM, TEMRA, Tex, CD4_T, B_cell, Myeloid, NK, Plasma; excluding Other/Unknown). Cells from responders and non-responders were extracted separately, and the R package “CellChat” was used to construct group-wise interaction networks. The data layer of the SCT assay was used as input (GetAssayData/LayerData, layer = “data”). After preprocessing, the R functions “identifyOverExpressedGenes” and “identifyOverExpressedInteractions” were applied. Communication probabilities were computed using the “trimean” method, and low-quality interactions were filtered (filterCommunication, min.cells = 10). Aggregated interaction networks were constructed via “aggregateNet”, and network centrality was computed using “netAnalysis_computeCentrality” (slot.name = “netP”). Interaction numbers and strengths were compared between the two groups (compareInteractions, measure = c(“count”, “weight”)), and differential interaction heatmaps (responder vs. non-responder difference matrices) were generated, focusing on Spark-like T-related interactions. The R function “netVisual_circle” was used to render Circos plots showing overall interactions, and “netVisual_bubble” was used to display ligand-receptor pairs for which Spark-like T cells served as the source (sources.use = “Spark-like_T”, targets.use = c(“Myeloid”, “CD4_T”, “B_cell”, “NK”, “TEM”, “Tex”, “TEMRA”), comparison = c(1,2)). The R algorithm “rankNet” (mode = “comparison”, stacked = TRUE) was used to compare differences in signaling pathway strength, and the “netAnalysis_signalingRole_heatmap” function was employed to display the characteristics of outgoing and incoming signaling. The top L-R pairs for Spark-like T cells were saved as SparkT_LR_pairs_Responder.csv and SparkT_LR_pairs_Nonresponder.csv.

### 2.7 Multi-source target integration and in silico knockout validation

The aforementioned analytical results, were integrated to construct a target gene set derived from multiple sources and comprising Spark Core signature genes, the top 50 differentially expressed genes (DEGs) between Spark-like T cells and other cells, the top 50 Monocle 3 trajectory genes, CellChat L-R gene pairs (ligand + receptor), key signaling pathway genes (including IFNG, STAT1, PRF1, GZMB, PDCD1, IL2, CCL5, TNF, and TRAF1), and prognostic genes (p < 0.05).

In silico knockout was performed using manifold inference: the top 3000 highly variable genes were selected to construct a PCA manifold, and a k-NN graph was constructed based on FNN; Gaussian high-dimensional distance weights were computed (weight = 1/(dist + 0.01)). For single-gene knockout, each cell’s k neighbors were queried, and the knockout contribution was inferred as a weighted average of neighboring cells’ expression after target gene removal. For combined knockout, the top 3 core genes (ranked by delta_pct and cor_to_module) were simultaneously knocked out in a multi-gene simulation. The percentage change in Spark Core module scores before and after knockout was calculated (Δ mean % = (ko_mean − wt_mean)/abs(wt_mean) + 1e-10) × 100), and Wilcoxon paired-test p-values were computed.

A target co-expression network was further constructed: the top 3000 highly variable genes in Spark-like T cells were extracted, Pearson correlation matrices were computed, and strongly co-expressed pairs with |correlation| > 0.3 were selected. The R packages “igraph” and “ggraph” were used to render the network with the top 5 hub genes and top 10 co-expressed genes. GO-BP/KEGG enrichment analysis was performed on the integrated hub target genes. A heatmap of percentage changes across multiple modules after combined knockout, including Cytotoxic, Exhaustion, T_cell_activ, Chemokine, and TNF_signaling was generated using the “pheatmap” package.

### 2.8 Spatial transcriptomics identification of Spark-like T cell distribution

Spatial analysis was performed based on GSE271689 GeoMx DSP data. PKC probe annotation files were read using the R function “jsonlite::fromJSON”, and the unique probe identifier RTS_ID was mapped to target gene names. DCC.gz raw count files were read, the “Code_Summary” block was extracted, and probe-ROI count matrices were constructed with RTS_ID as row names using the “FindMarkers” package. Probe-level expression was aggregated to gene-level expression using the “aggregate” function, and Seurat objects were constructed with thresholds set at min.cells = 3 and min.features = 100, followed by “NormalizeData”, “FindVariableFeatures”, “ScaleData”, PCA (dims = 1:20), “FindNeighbors” (dims = 1:20), “FindClusters” (resolution = 0.5), and “RunUMAP” processing.

The full GSE207422 dataset system was used as the RCTD reference, and the “spacexr::create.RCTD” function was applied for deconvolution to obtain cell type proportions for each ROI. Spark T scores were computed for each ROI based on Spark T signature genes. ROIs with significant differences in cell type proportions between responders and non-responders were identified using the “FindMarkers” function with the threshold set at min.pct = 0.1 and logfc.threshold = 0.25. Marker expression bubble plots for each cluster were generated using the “ggplot2” package, and RCTD deconvolution accuracy was verified.

Spark T scores across different spatial clusters were analyzed using the “ggplot2” package. Differentially expressed genes (DEGs) between regions with high and low Spark T scores regions were identified using “FindMarkers”, followed by GO-BP enrichment using the “enrichGO” function. Spatial co-localization of Spark-like T cells with other cell types was assessed via Spearman’s correlation analysis, and positively correlated partners were selected using r > 0.1 and p < 0.05. Co-localization scatter plots were generated (geom_smooth, method = “lm”), and characteristic gene module correlation validation was performed.

### 2.9 Construction of the Spark-like T cell-related prognostic signature

A 14-gene Spark-like T cell-related prognostic signature was constructed on the basis of Spark-like T cell marker genes. First, 53 prognosis-associated DEGs were identified through univariate Cox regression analysis in the training cohort TCGA-NSCLC. Subsequently, LASSO penalized Cox regression analysis was applied to minimize the risk of overfitting using the R package “glmnet”, and a 14-gene model was constructed. Patient risk scores were calculated using the expression levels of the 14 genes and their corresponding regression coefficients:

In this formula, “n” represents the number of genes; “expi” represents the expression level of gene “i”; and “βi” represents the coefficient of gene “i”. Patients were divided into high-risk and low-risk groups according to the median risk score, and OS was analyzed in both groups using the R package “survminer”. The “survminer” and “timeROC” packages were used to perform time-dependent ROC curve analysis to assess the predictive efficacy of the prognostic models. Finally, risk scores were calculated in the validation cohorts GSE37745, GSE41271 and GSE72094 using the same formula.

### 2.10 Functional enrichment analysis for the NSCLC samples

Enrichment analyses for KEGG pathways, oncogenic signatures, and gene sets from the Curated Cancer Cell Atlas were performed using the GSVA algorithm to calculate a score for each pathway in each sample. Differentially activated pathways between the high- and low-risk score groups were identified using the “limma” package, using FDR < 0.05 as the significance threshold. Differentially activated KEGG pathways between the high- and low-risk score groups were analyzed using the gene set enrichment analysis (GSEA) algorithm.

### 2.11 Estimation of immune cell infiltration in the TME

The “CIBERSORT” algorithm implemented in the R package “IOBR” was used to estimate the abundance of 22 immune cell types in samples from the TCGA-NSCLC cohort. This algorithm uses gene expression profiles to statistically estimate the infiltration proportions of cell subtypes in complex tissues and thereby assess the abundance of specific cell types in mixed tissues. Immune and stromal scores were calculated using the “ESTIMATE” algorithm based on bulk transcriptomic data. The immune score reflects the inferred infiltration level of immune cells in tumor tissues, whereas the stromal score reflects the inferred abundance of stromal components. Higher scores indicate relatively higher immune or stromal content within the tumor microenvironment. These scores are computational estimates based on bulk RNA-seq profiles rather than direct cell-counting measurements.

### 2.12 Statistical analysis

All analyses were performed using R software (v4.3.0). Comparisons of continuous variables between two groups were performed using the Wilcoxon rank-sum test or independent-sample t test. Multi-group comparisons employed the Kruskal-Wallis test. Correlation analysis used Spearman’s rank correlation. Associations between categorical variables were tested using Fisher’s exact test. DEGs were considered significant at Benjamini-Hochberg false discovery rate (FDR)-corrected p-values (p_val_adj) < 0.05. Differences in module scores after in silico knockout were assessed using the Wilcoxon paired tests, and permutation test were used to compare knockouts of actual targets with null distributions generated by random gene knockouts. In spatial analysis, Cliff’s Delta was used to evaluate effect sizes (|d| > 0.33 for small effect, > 0.474 for medium effect, > 0.741 for large effect). Statistical significance was set at a two-tailed p < 0.05. For graphical visualization, statistical significance was annotated as follows: ns, not significant (p > 0.05); *p < 0.05; **p < 0.01; ***p < 0.001. For prognostic assessment, survival curves were constructed using the Kaplan-Meier method, and between-group differences were assessed using the log-rank test.

## 3 Results

### 3.1 Construction of single-cell atlas and heterogeneity analysis of the TME in patients with NSCLC treated with anti-PD-1 immunotherapy

The overall flow chart of this study is presented in **Figure 1**. To systematically characterize the cellular heterogeneity of the tumor microenvironment and its association with treatment response in NSCLC patients receiving anti-PD-1 immunotherapy, we first constructed a single-cell atlas based on the post-treatment GSE207422 dataset, which contains surgical specimens collected after neoadjuvant anti-PD-1-based therapy. The data underwent rigorous preprocessing and quality control. After standard filtering, the number of genes detected, total UMI counts, and mitochondrial gene percentages across all samples met downstream analytical criteria (**Supplementary Figure 1A**). PCA was used to assess data dimensionality, and the first 30 principal components were selected to capture the major biological variation (**Supplementary Figure 1B**). The Harmony algorithm effectively corrected inter-sample batch effects, ensuring that cell clustering was not driven by technical factors (**Supplementary Figure 1C**). Using shared nearest neighbor (SNN) graph clustering and multi-resolution evaluation (**Supplementary Figures 1D, E**), 36 transcriptionally distinct cell clusters were obtained at a resolution of 0.8 (**Supplementary Figure 2A**). Based on canonical lineage marker expression patterns, these cell clusters were robustly annotated as 9 major cell types, namely epithelial cells, CD8^+^T cells, CD4^+^T cells, B cells, myeloid cells, mast cells, fibroblasts, NK cells, and plasma cells (**Supplementary Figures 2B–D**).

**Figure 1:**
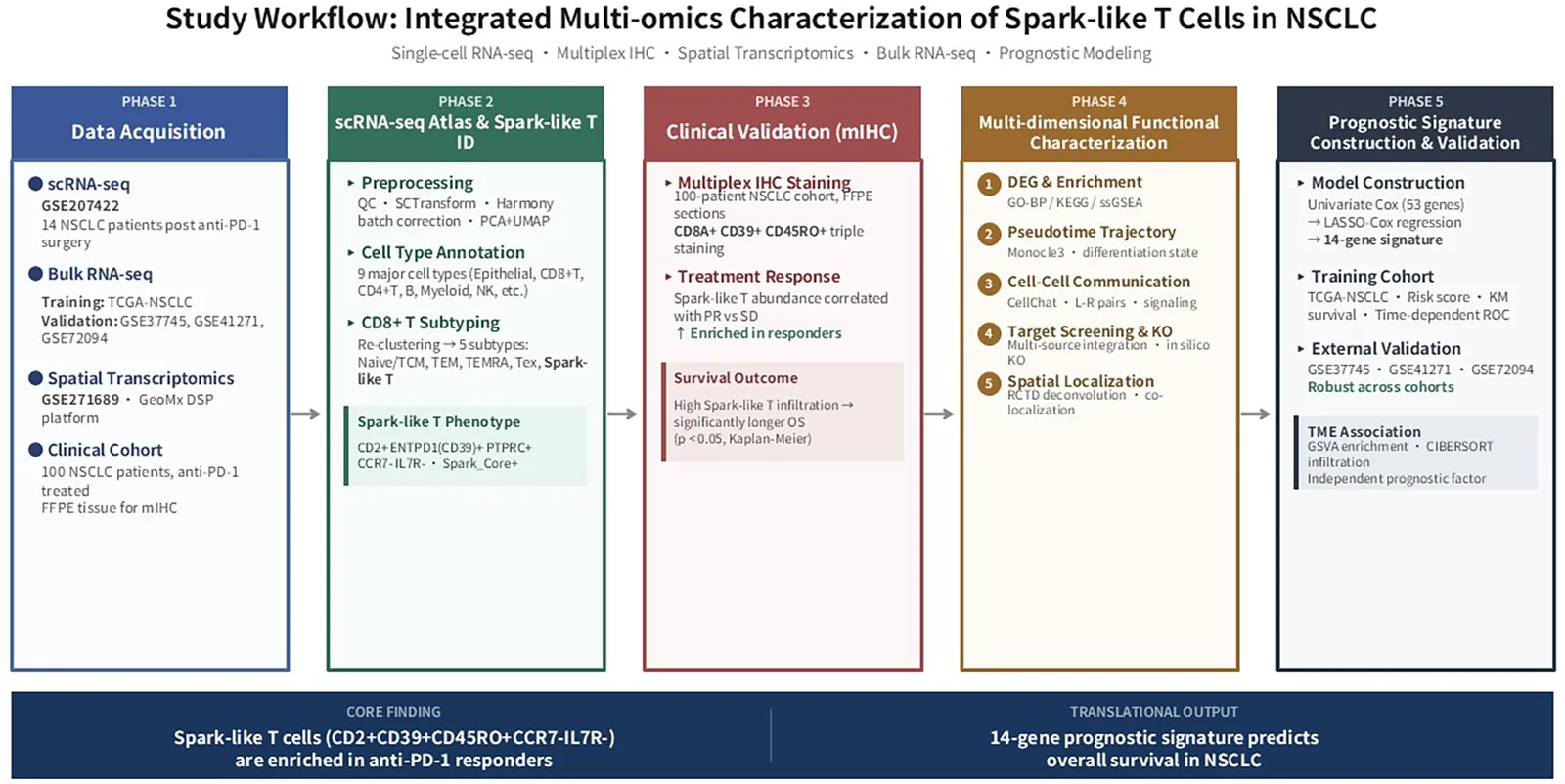
The overall flow chart of this study.

We subsequently compared cellular composition between responders and non-responders to anti-PD-1 immunotherapy. UMAP projections indicated markedly distinct spatial distribution patterns of cells between the two groups (**Supplementary Figure 2B**), and cell type composition across individual samples differed significantly between groups (**Supplementary Figure 2E**). Quantitative analysis indicated that the proportion of infiltrating CD8^+^T cells infiltration in tumor tissues was significantly higher in responders than in non-responders (**Supplementary Figures 3A–C**; **Supplementary Table 1**), suggesting a potential association between CD8^+^T cell abundance and anti-PD-1 immunotherapeutic efficacy.

To characterize the molecular features of CD8^+^T cells associated with treatment response, differential expression analysis was performed between the two groups and identified significant transcriptomic differences (**Supplementary Figures 3D, F**; **Supplementary Table 1**). Functional enrichment analysis suggested that genes upregulated in responders were significantly enriched in biological processes such as lymphocyte proliferation, mononuclear cell proliferation, and regulation of leukocyte proliferation. In contrast, genes downregulated in responders were significantly enriched in cell-cell adhesion regulation, viral defense response, immune response activation, and T cell activation regulation pathways (**Supplementary Figure 3E**; **Supplementary Table 1**). KEGG pathway analysis further indicated that these differentially expressed genes were closely associated with viral infection-related pathways and Th17 cell differentiation (**Supplementary Figure 3G**; **Supplementary Table 1**). GSEA suggested that embryonic development, cell death regulation, and apoptosis-related gene sets were significantly suppressed in CD8^+^T cells from responders, as were pathways related to transcriptional dysregulation in cancer (**Supplementary Figure 3H**).

### 3.2 Identification of tumor-infiltrating CD8^+^T cell functional states and molecular characterization of Spark-like T cells

The differential expression and pathway analyses described above not only confirmed global transcriptomic remodeling of CD8^+^T cells across treatment response groups, but also provided a direct rationale for focusing on a specific functional program: the genes and biological processes upregulated in CD8^+^T cells from responders, including lymphocyte proliferation and activation programs, converged on the core effector and immunoregulatory signature recently defined for Spark T cells (**Supplementary Figures 3D–H**). We therefore hypothesized that the CD8^+^T cell population defined by this Spark T cell transcriptional program, rather than any of the other functional states, would be the population most closely associated with anti-PD-1 response. To test this hypothesis, we performed unsupervised re-clustering of tumor-infiltrating CD8^+^T cells and applied a literature-based, hypothesis-driven gating strategy (Section 2.3) to identify Spark-like T cells among the resulting populations. CD8^+^T cells were stably partitioned into 5 functional states: naive/central memory T cells (Naive/TCM), effector memory T cells (TEM), terminally differentiated effector memory T cells (TEMRA), exhausted T cells (Tex), and Spark-like T cells with core transcriptional features (**Figures 2A, B**). UMAP projections and violin plots suggested that among the canonical markers of Spark-like T cells, CD2, ENTPD1 (CD39), and PTPRC (CD45) were significantly up-regulated in Spark-like T cells compared with other states of CD8^+^T cells, while CCR7 and IL7R were considerably down-regulated in Spark-like T cells (**Figure 2C**). This expression pattern was consistent with a recently published finding (10). Spark T core module scoring showed that Spark-like T cell-related clusters were specifically enriched for this module (**Figure 2D**).

**Figure 2:**
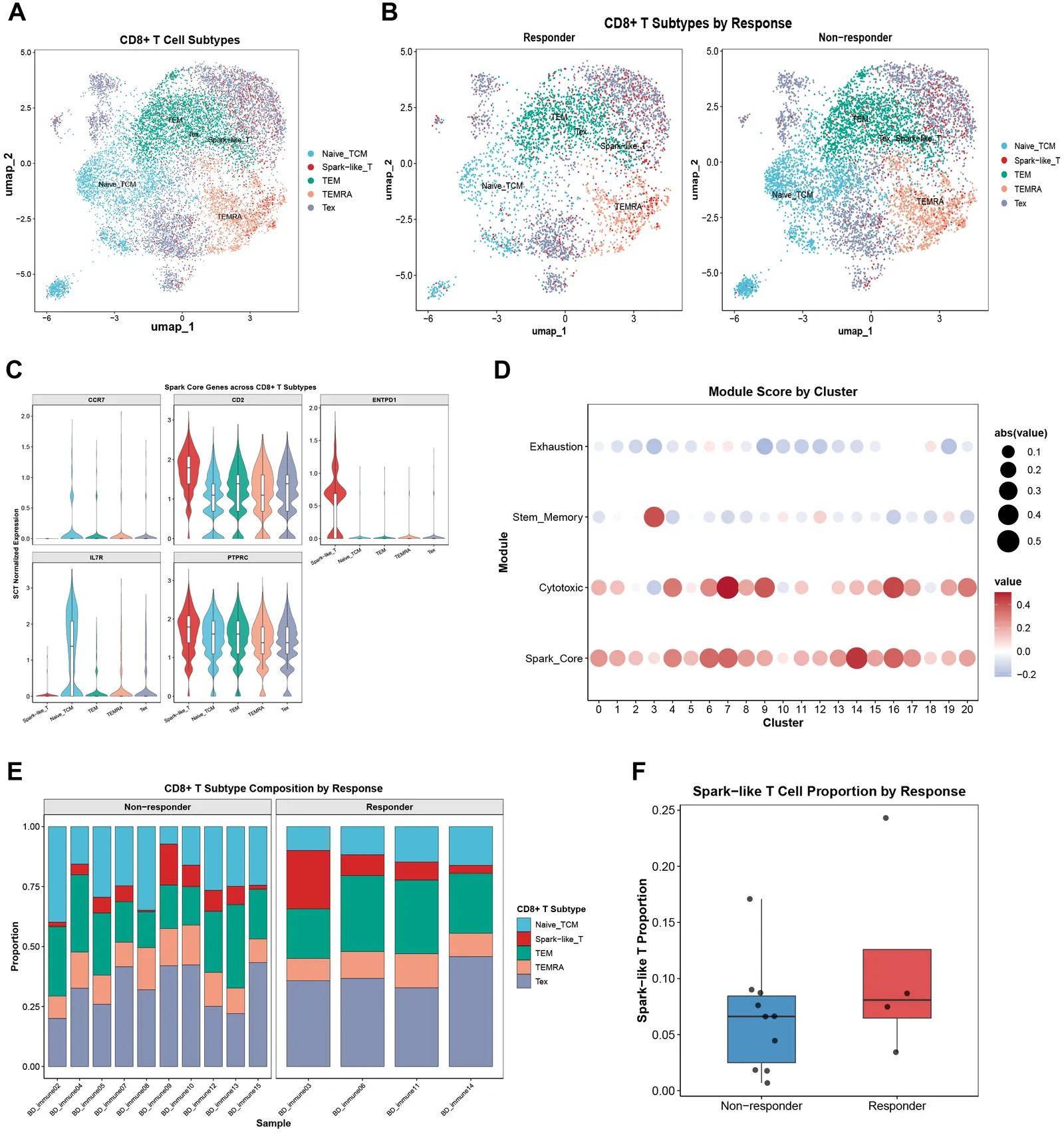
Identification of tumor-infiltrating CD8^+^T cell populations and their distribution characteristics across different immunotherapeutic response groups. **(A)** UMAP plot of unsupervised clustering showing the 5 populations of CD8^+^T cells. Different colors represent different CD8^+^T cell populations, and the names of the 5 populations are annotated on the right. **(B)** UMAP plots showing the distribution of different CD8^+^T cell populations in the clinical samples from responders and non-responders. Different colors represent different CD8^+^T cell populations, and the names of the 5 populations are annotated on the right. **(C)** Violin plots showing the expression levels of Spark-like T cell marker genes (CCR7, CD2, ENTPD1, IL7R, and PTPRC) in different CD8^+^T cell populations. Different colors represent different CD8^+^T cell populations. The names of the 5 populations are listed in the vertical axis. **(D)** Module score distribution of Spark T core, Cytotoxic, Stem_Memory, and Exhaustion gene sets across CD8^+^T cell clusters. Each dot represents a cluster, with dot size indicating the absolute average module score and color reflecting the sign and magnitude of the scor. Clusters 4, 6, 7, 14, and 16 exhibit notably high Spark_Core module scores, supporting their classification as Spark-like T cells. **(E)** Stacked bar charts showing the proportion of CD8^+^T cell populations in samples from responders and non-responders. Different colors represent different CD8^+^T cell populations. The grouping status is indicated on the top, and the names of each sample is indicated in the vertical axis of the chart. **(F)** Box plots comparing the proportion of Spark-like T cells between responders and non-responders. Red represents responder group while blue represents non-responder group. The group names are indicated in the vertical axis of the plot.

To investigate the association between each population and immunotherapeutic response in patients with NSCLC, we compared population composition between anti-PD-1 treatment response groups. UMAP projections showed that Spark-like T cells showed a significant trend toward enrichment in the responder group (**Figures 2B, E, F**). Quantitative analysis further suggested that Naive-TCM and Tex states predominated in non-responders, whereas the proportions of Spark-like T cells and TEM were significantly higher in the responders (**Figures 2B, E, F**), suggesting that Spark-like T cell enrichment is positively correlated with anti-PD-1 treatment response.

As shown in the UMAP projection, Spark-like T cells overlap with adjacent CD8^+^T cell populations, consistent with a transcriptional/functional state embedded within the CD8^+^T cell continuum. Nevertheless, this state exhibited a reproducible transcriptional signature, as reflected by the differential expression of its defining markers (**Figure 2C**) and the specific enrichment of the Spark_Core module (**Figure 2D**). Given that its identification relies on a predefined marker panel and module-score thresholds (Section 2.3), we interpret Spark-like T cells as a definable functional state rather than a fully segregated lineage, and refer to this population as a “Spark-like T cell state/population”.

### 3.3 Validating the association between Spark-like T cells and clinical outcomes of patients with NSCLC receiving immunotherapy using mIHC

To further investigate whether the transcriptomically defined Spark-like T cells could potentially be used to predict clinical outcomes in NSCLC patients treated with anti-PD-1 immunotherapy, we performed mIHC using a surrogate immunophenotype, CD8A^+^CD39^+^CD45RO^+^, in a 100-sample clinical cohort. Patient clinical characteristics are presented in **Table 1** and **Supplementary Table 2**. Representative staining images from patients with PR and SD are shown in **Figures 3A, B**. First, we analyzed the associations between Spark-like T cell intensity in tumor tissues and patient clinical features including T stage, N stage, and clinical stage. The results indicated patients with advanced T stage, N stage, and clinical stage had lower Spark-like T cell infiltration in their tumors (**Figures 3C–E**). Notably, patients with PR to anti-PD-1 immunotherapy had significantly greater Spark-like T cell intensity in their tumor tissues (**Figure 3F**). We also analyzed the association between Spark-like T cell intensity and patients’ prognosis. First, we analyzed the relationship between T stage, N stage, clinical stage, and overall survival (OS) of the 100 patients to examine whether there are deviations in the clinical information of the collected samples. The results showed that patients tended to have shorter OS with advancing T stage, N stage, and clinical stage (**Figures 3G–I**), suggesting that the clinical information from the 100 collected samples showed no deviation. Importantly, patients with high Spark-like T cell intensity in their tumor tissues had significantly longer OS compared with those with lower Spark-like T cell infiltration (**Figure 3J**). The association between Spark-like T cell abundance and survival was examined across different tumor stages. The results showed that among patients with the same T, N, and clinical stage, those whose tumor tissues with a high infiltration of Spark-like T cells still tended to have a better prognosis (**Figures 4A–I**). Moreover, the results of multivariable Cox regression analysis also indicated that high infiltration of Spark-like T cells was a protective factor independent of disease stages and PD-L1 tumor proportion score (TPS) for the prognosis of patients with NSCLC (**Figure 4J**). Although the proportion of patients showing PR to immunotherapy was higher in the group with PD-L1 TPS > 1% compared with the group with PD-L1 TPS < 1% (56% vs 17%) (**Supplementary Figure 4A**), PDL1 TPS was not an independent factor for the prognosis of NSCLC patients (**Figure 4J**). These results suggested that the CD8A^+^CD39^+^CD45RO^+^ surrogate immunophenotype, which is consistent with the transcriptomically defined Spark-like T cell state, may serve as a biomarker associated with anti-PD-1 therapeutic efficacy and survival time in patients with NSCLC.

**Figure 3:**
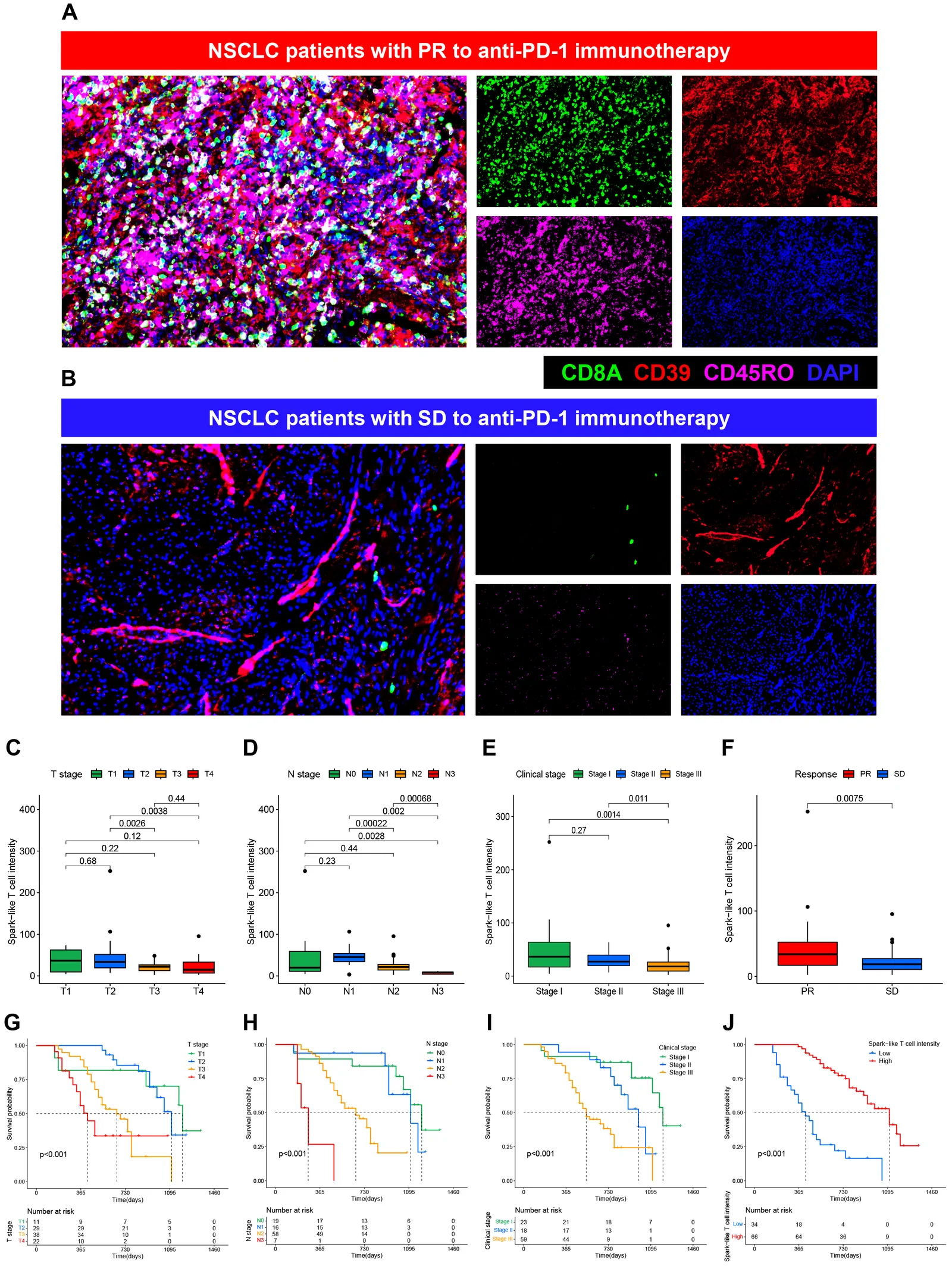
The results of mIHC assay suggesting that NSCLC patients with PR to anti-PD-1 immunotherapy had higher Spark-like T cells infiltration in their tumor tissues. **(A)** Representative immunofluorescence staining image of Spark-like T cell markers CD8A, CD39, and CD45RO in the tumor tissues of patients with PR to anti-PD-1 immunotherapy. **(B)** Representative immunofluorescence staining image of Spark-like T cell markers CD8A, CD39, and CD45RO in the tumor tissues of patients with SD to anti-PD-1 immunotherapy. **(C–F)** Box plots showing the intensity of Spark-like T cells in the tumor tissues of NSCLC patients with different **(C)** T stages, **(D)** N stages, **(E)** clinical stages, and **(F)** response to anti-PD-1 immunotherapy. In each box plot, the grouping status is marked on the abscissa axis, and the vertical axis represent Spark-like T cell infiltration levels. **(G–J)** Kaplan-Meier survival curves reflecting the overall survival times of NSCLC patients with different **(G)** T stages, **(H)** N stages, **(I)** clinical stages, and **(J)** Spark-like T cell intensity. The abscissa axis shows survival time while the ordinate axis shows survival probability. Different colors represent different groups. The numbers of patients in each group are indicated at the bottom of the chart. PR, partial response; SD, stable disease.

**Figure 4:**
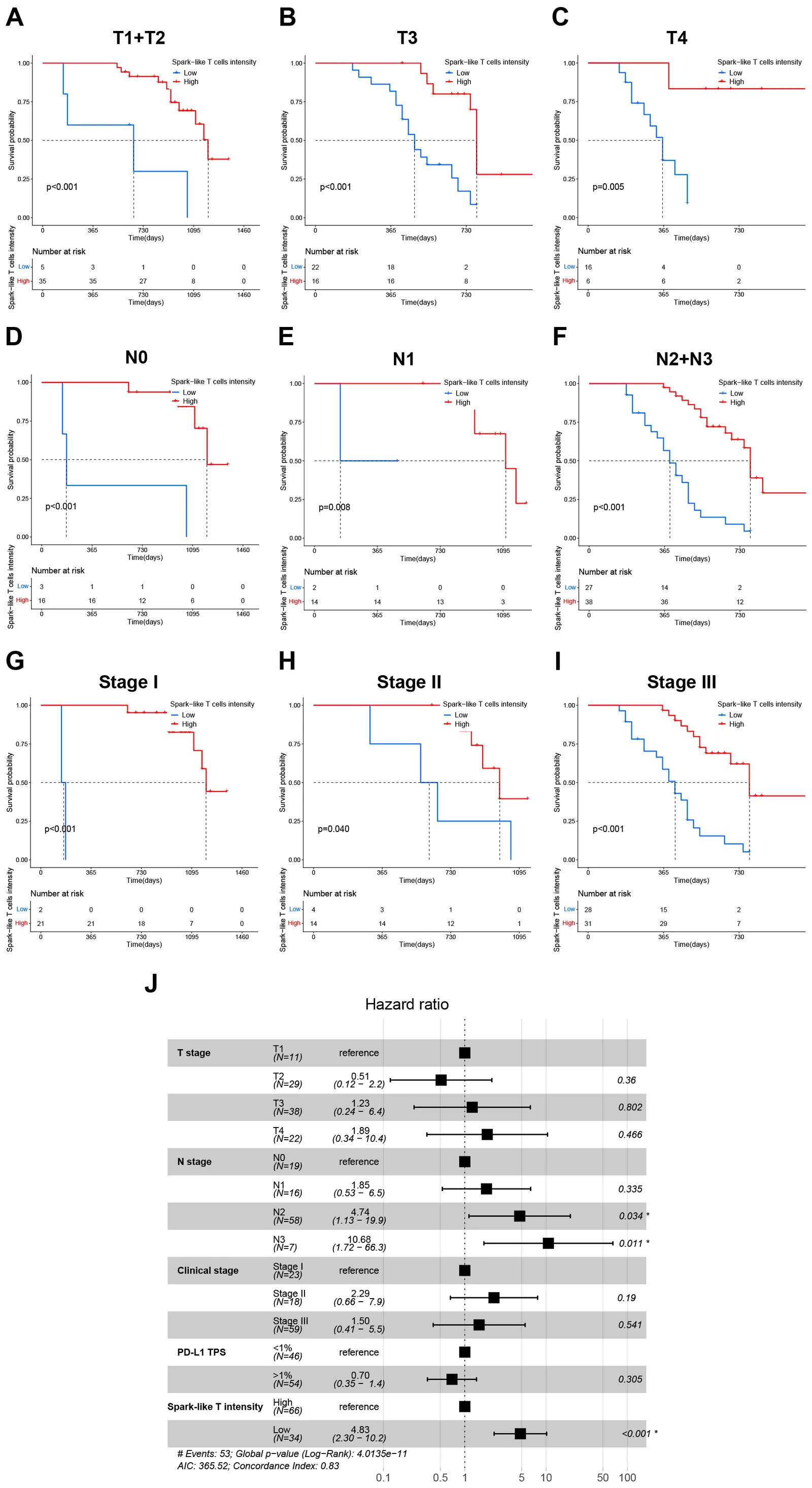
The association between Spark-like T cell abundance and the prognosis of NSCLC patients is independent of disease stages and TPS in the mIHC cohort. **(A-I)** Kaplan-Meier survival curves showing the overall survival times of NSCLC patients with low- and high-infiltration of Spark-like T cells and **(A)** T1 or T2 stage, **(B)** T3 stage, **(C)** T4 stage, **(D)** N0 stage, **(E)** N1 stage, **(F)** N2 or N3 stage, **(G)** clinical stage I, **(H)** clinical stage II, and **(I)** clinical stage III. The abscissa axis shows survival time while the ordinate axis shows survival probability. Different colors represent different groups. The numbers of patients in each group are indicated at the bottom of the chart. **(J)** Forest plot showing that Spark-like T cell intensity was an independent prognostic factor through multivariate Cox regression analysis in the mIHC cohort. The names of the variables are on the left of the plot. The hazard ratio of each variable and the corresponding forest plot are in the middle of the plot. The p-values of the corresponding variables are on the right of the plot. TPS, tumor proportion score.

### 3.4 Functional enrichment analysis for the molecular features of Spark-like T cells

To further elucidate the unique transcriptional program of Spark-like T cells, we identified specific marker genes for each population and found that Spark-like T cells specifically expressed core effector and immunoregulatory genes including GNLY, GZMB, LAG3, HAVCR2, ENTPD1, and CXCL13 (**Supplementary Table 3**). The co-expression of effector (GNLY, GZMB) and antigen-experience/exhaustion-associated (LAG3, HAVCR2, ENTPD1, CXCL13) genes in Spark-like T cells is consistent with an intermediate state within the exhaustion-differentiation continuum rather than with a terminally exhausted phenotype (11). TEMRA was enriched for terminal differentiation-related genes such as FASLG, IFNG, CCL3, TNF, and ZFP36L1. Naive-TCM was enriched for stemness-related genes including TCF7, IL7R, GPR183, and KLF2. Tex was enriched for effector memory-associated genes such as CST7, GZMK, and SLAMF7 (**Supplementary Figure 5A**). Notably, although some exhaustion-associated genes, such as LAG3 and HAVCR2 were also expressed in Spark-like T cells, they were co-expressed with core effector and immunoregulatory genes including GNLY, GZMB, and ENTPD1. The subtype annotation was based on the overall marker program and multi-module scoring rather than on individual marker genes, and Spark-like T cell clusters exhibited high Spark_Core module scores without a dominant exhaustion module, whereas Tex clusters showed the opposite pattern (**Figure 2D**). In addition, pseudotime analysis placed Spark-like T cells at the mid-trajectory transition region, distinct from the terminally exhausted compartment (**Supplementary Figures 6A, B**), further supporting the separation between these two populations. GO-BP enrichment analysis suggested that Spark-like T cell-specific marker genes were significantly enriched in pathways related to immune activation, such as regulation of T cell activation, positive regulation of cytokine production, and T cell differentiation (**Supplementary Figure 5B**). KEGG enrichment analysis further suggested their significant involvement in cell adhesion molecule (CAM) interaction (**Supplementary Figure 5C**). Specifically, the ssGSEA results indicated significant differences in multiple immune response-related pathways between non-responder and responder groups: vaccine response-related pathways (such as QUEREC_PBMC_YF_17D_VACCINE, ZAK_PBMC_MRKAD5_HIV_1_GAG_POL_NEF) and antiviral immune pathways were significantly downregulated in the responder group, whereas they were highly activated in non-responders (**Supplementary Figure 5D**; **Supplementary Table 4**). In addition, epithelial-mesenchymal transition (such as HALLMARK_EPITHELIAL_MESENCHYMAL_TRANSITION) and NK cell-related pathways (such as HOEK_NK_CELL_2011_2012_TIV_ADULT_1DY_UP) showed different distributions between the two groups (**Supplementary Figure 5D**; **Supplementary Table 4**). These results suggested that Spark-like T cells may regulate anti-PD-1 immunotherapeutic response through these biological pathways in the TME.

### 3.5 Pseudotime differentiation trajectory of Spark-like T cells and association with treatment response

Based on the identified CD8^+^T cell functional states, we employed Monocle3 pseudotime analysis to investigate the developmental origin and hierarchical position of Spark-like T cells. Unsupervised trajectory inference suggested that CD8^+^T cells exhibited a continuous developmental path from an initial/central memory state (Naive/TCM, low pseudotime) to terminal effector and exhausted states (Tex, high pseudotime) (**Supplementary Figure 6A**). Spark-like T cells were predominantly enriched in the mid-trajectory and early transition regions, suggesting that they may represent a unique T cell state at the transition between acquisition of effector function and differentiation (**Supplementary Figure 6B**). Notably, Spark-like T cells were rarely detected at the terminal end of the trajectory corresponding to terminally differentiated/exhausted cells, arguing against a terminally exhausted identity and supporting their positioning as an intermediate, antigen-experienced transitional state (**Supplementary Figure 6B**).

To investigate whether treatment response remodels the differentiation program of Spark-like T cells, we compared trajectory distributions between responders and non-responders. The results showed that the distribution of cells along pseudotime differed significantly between the two groups (**Supplementary Figure 6C**). Quantitative analysis suggested that pseudotime values of Spark-like T cells were higher overall in non-responders than in responders (**Supplementary Figure 6F**), indicating that Spark-like T cells in non-responders may occupy a more advanced or terminally differentiated state.

We further identified gene modules with significant dynamic changes along the differentiation trajectory. Pseudotime-dependent gene box heatmaps suggested that responders and non-responders exhibited markedly distinct gene expression programs during differentiation (**Supplementary Figure 6D**). Analysis of key marker gene expression along pseudotime showed that expression of the stemness maintenance genes CCR7 and IL7R decreased with advancing pseudotime, whereas ENTPD1 was highly expressed in the mid-region corresponding to Spark-like T cells, while CD2 and PTPRC, as common T cell markers, were widely distributed across differentiation stages (**Supplementary Figure 6E**). These results collectively indicate that the differentiation state of Spark-like T cells is associated with ICB treatment response, and the tendency toward terminal differentiation in this population among non-responders may be related to functional exhaustion and treatment resistance.

### 3.6 Differential remodeling of Spark-like T cell interaction networks across treatment response groups

Building on the analysis of Spark-like T cell differentiation trajectories, we further employed the CellChat algorithm to construct cell interaction networks for responder and non-responder groups to systematically characterize differences in communication between Spark-like T cells and other immune and non-immune cells in the TME and their association with treatment response. Overall interaction statistics showed differences in both total interaction number and strength between the responder and non-responder groups, indicating that the overall characteristics of cell communication networks differ across treatment response groups (**Supplementary Figure 7A**). The heatmap of differences in interaction numbers showed significant heterogeneity in interaction counts for specific cell type pairs between the two groups. For example, the number of interactions between Spark-like T cells and myeloid cells decreased by 7 in the responder group compared with the non-responder group, while interactions with Naive_TCM increased by 6, reflecting the reorganization of intercellular communication networks across treatment response groups (**Supplementary Figure 7B**). The heatmap of differences in interaction strength further suggested systematic differences in communication weights across multiple cell type pairs: interaction strengths between Naive_TCM and B_cell, CD4_T, NK, and TEM cells were significantly enhanced in the responder group, while interaction strengths between myeloid and Tex, TEM, Spark-like_T, and NK cells showed a decreasing trend (**Supplementary Figure 7C**). Cell interaction network graphs intuitively displayed the interaction topology of Spark-like T cells with CD4_T, B_cell, NK, TEM, Tex, TEMRA, Naive_TCM, and plasma subtypes in both responder and non-responder groups (**Supplementary Figures 7D, E**). In responders, interaction lines between Spark-like T cells and various immune cells were denser and thicker, suggesting a predicted more active communication network. Ligand-receptor pair analysis identified specific molecular pairs differentially expressed between Spark-like T cells and B_cell, CD4_T, Myeloid, NK, TEM, TEMRA, and Tex subpopulations, involving HLA family molecules, CCL chemokines, IFNG, GZMA, CXCL13, CD99, and PTPRC, among other immunoregulatory communication pathways (**Supplementary Figure 7F**). Comparison of signaling pathway strength showed that the MHC-I pathway was dominant in both groups, and the relative contributions of MHC-I, MHC-II, CCL, CD99, GALECTIN, PARs, and LCK pathways were slightly higher in the responder group than in the non-responder group (**Supplementary Figure 7G**), further suggesting that Spark-like T cells were associated with antitumor immune features and increased positive immune signaling pathways in responders. It should be noted that CellChat infers potential ligand-receptor communication from gene expression patterns. These results therefore represent predicted communication differences and do not demonstrate signaling flux, causal directionality, or functional enhancement of antitumor immunity.

Further analysis of signaling patterns indicated that in the responder group, signaling pathways including MHC-I, MHC-II, CCL, CD99, GALECTIN, PARs, CD45, and LCK exhibited prominent outgoing signal patterns in Spark-like T cells and other immune cell subtypes (**Supplementary Figure 8A**), whereas these cell subtypes simultaneously received incoming signals from MHC-I, MHC-II, GALECTIN, CD45, FN1, and IL16 pathways (**Supplementary Figure 8B**), suggesting that Spark-like T cells may participate in complex immunoregulatory networks through predicted bidirectional functional ligand-receptor interactions in responders. Cell interaction network graphs further displayed the communication relationships among cell subtypes (**Supplementary Figures 8C, D**). To evaluate whether the 14-gene signature provides incremental prognostic information beyond established clinical variables, we compared its discriminative performance with clinical features including age, sex, and clinical stage using ROC analysis. In most cohorts, the risk score model exhibited higher AUCs compared with individual clinical variables including clinical stage (**Supplementary Figures 11A–D**).

### 3.7 Multi-dimensional screening and in silico knockout validation of key targets in Spark-like T cells

Based on the aforementioned multi-dimensional analyses, we integrated DEGs, pseudotime trajectory (Trajectory), ligand-receptor interactions (LR), functional pathways (Pathway), and prognostic information to systematically screen core regulatory targets of Spark-like T cells (**Supplementary Table 5**). The integrated target contribution ranking showed that mitochondrially encoded genes (MT-ND2, MT-CO1, MT-CO2, MT-ATP6, MT-CO3) and immune-related genes (CCL3, HLA-DPA1, HLA-DRB1, HSPA1A) had the strongest correlations with the integrated modules (**Figure 5A**). The integrated target co-expression network indicated that TNF, CLDND2, and HSPA1B served as core nodes within coordinated regulatory networks (**Figure 5C**). GO-BP enrichment analysis indicated that integrated target genes were significantly enriched in immune processes including leukocyte-cell adhesion, T cell activation regulation, and lymphocyte-mediated immunity (**Figure 5D**). KEGG pathway enrichment analysis further indicated their involvement in cell adhesion molecule interactions, antigen processing and presentation, and multiple viral infection-related pathways (**Figure 5E**). To validate the functional necessity of core genes, we employed the scTenifoldKnk-style virtual knockout (KO) strategy to perform single-gene (TNF, ENTPD1, IL2, IFNG, GZMB, RUNX3) and multi-gene combined KO in the scRNA-seq data. The results showed that the KO conditions had varying effects on Spark Core module scores (**Figure 5B**; **Supplementary Table 6**). Further analysis of functional module changes after combined TNF+ENTPD1+IL2 KO suggested a downward trend in Chemokine, Exhaustion, T_cell_activ, TNF_signaling, Spark_Core, and Cytotoxic pathways at the single-cell level, with the Chemokine and Exhaustion modules showing the most pronounced downregulation (**Figure 5F**).

**Figure 5:**
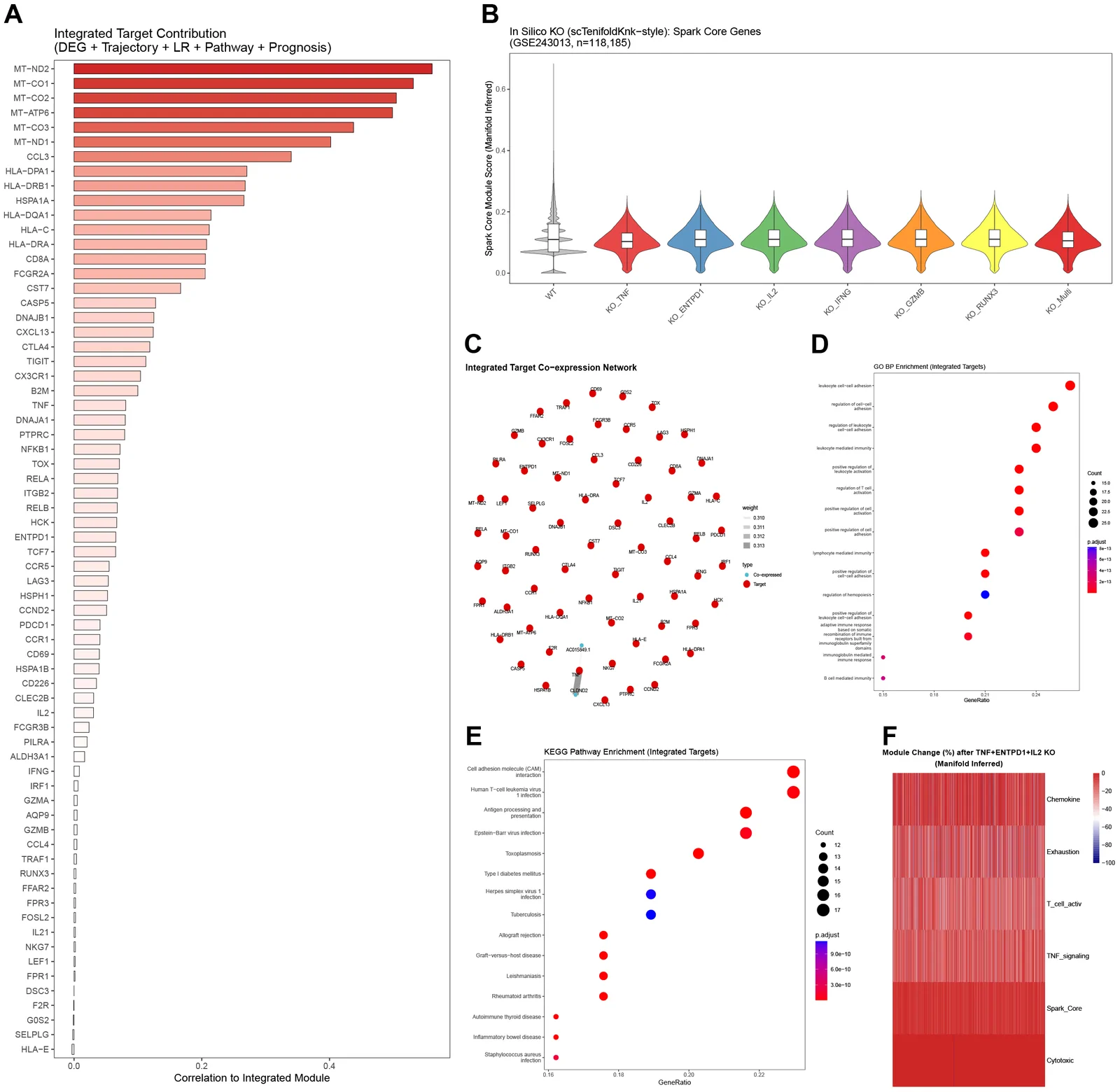
Integrated screening, functional enrichment, and virtual KO validation of core target genes for Spark-like T cells. **(A)** Ranked contribution of candidate target genes based on five evidence sources: differentially expressed genes (DEGs), pseudotime trajectory, ligand-receptor (LR) interactions, signaling pathways, and prognosis. The gene names are listed on the abscissa axis, and the vertical axis represents the correlation coefficient of each gene. **(B)** Violin plots showing Spark_Core module scores under wild-type (WT) and single- or multi-gene virtual knockout (KO) conditions in GSE243013 (n = 118,185 cells). The vertical axis indicates groups with different gene KO, and the vertical axis indicates Spark core module score. **(C)** Co-expression network of integrated target genes. Each dot represents one gene. **(D, E)** Bubble plots of **(D)** GO-BP and **(E)** KEGG enrichment analysis for target genes. The names of the biological processes are shown on the abscissa axis, and the gene ratios are indicated on the vertical axis. The sizes of the bubbles mean the number of gene enriched in each biological process. Red color of the dots means higher p-value, while blue means lower p-value. **(F)** Heatmap of percentage changes in Chemokine, Exhaustion, T_cell_activ, TNF_signaling, Spark_Core, and Cytotoxic module activities after combined TNF + ENTPD1 + IL2 KO. The name of each module is marked on the right of the plot.

### 3.8 Ecological niche analysis and microenvironmental co-localization of Spark-like T cells at the spatial transcriptome level

Based on spatial transcriptomics data, we mapped scRNA-seq cell type annotations onto spatial tissue coordinates using the RCTD deconvolution algorithm to systematically characterize the spatial distribution characteristics, regional heterogeneity, and co-localization of Spark-like T cells with neighboring immune cells in the TME. First, we validated the RCTD deconvolution results using canonical marker genes, which showed that marker genes for T_CD8 (CD8A, CD8B, CD3D, CD3E), T_CD4 (CD4, IL7R, FOXP3), B_cell (CD19, MS4A1, CD79A), NK (NKG7, GNLY, KLRD1, NCAM1), Macrophage_M1 (CD86, CD80, IL1B, TNF, IRF5, NOS2, CXCL9, CXCL10), myeloid (CD68, CD14, CD163, CSF1R, LYZ), mast (TPSAB1, CPA3, KIT), fibroblast (COL1A1, COL1A2, PDGFRA, ACTA2), epithelial (EPCAM, KRT5, KRT8, KRT18), and endothelial (PECAM1, VWF, CDH5) cells were specifically and highly expressed in the corresponding spatial clusters, confirming the accuracy of the deconvolution annotation (**Supplementary Figure 9A**). CD8^+^T cell spatial distribution analysis showed that the proportion of CD8^+^T cells estimated by RCTD increased progressively from Cluster 0 to Cluster 4 (**Supplementary Figure 9B**).

To delineate the spatial organization of Spark-like T cells within the TME, we applied RCTD deconvolution to spatial transcriptomics data and estimated Spark-like T cell proportions across five spatially defined clusters (**Figure 6A**). Spark-like T cell enrichment followed a pronounced gradient, with progressively higher proportions from cluster 0 through cluster 4, indicating substantial regional heterogeneity in their tissue distribution. Consistent with this, bubble plot analysis confirmed that clusters 3 and 4 harbored both higher mean Spark-like T proportions and elevated detection rates across ROIs, whereas clusters 0 and 1 were characterized by low Spark-like T abundance (**Figure 6B**). We next assessed the spatial co-localization of Spark-like T cells with other cellular compartments using Spearman correlation analysis (**Figure 6C**). Spark-like T cell proportions were significantly and positively correlated with mast cells, B cells, naive/central memory T cells, TEMRA cells, CD4^+^T cells, fibroblasts, and myeloid cells, suggesting that Spark-like T cells preferentially reside in immune- and stroma-rich niches. Whether this enrichment reflects a specific niche that actively promotes the Spark-like T cell state, or a passive consequence of an overall inflamed, immune- and stroma-rich microenvironment, cannot be distinguished at the ROI level with the present data. Conversely, significant negative correlations were observed with epithelial cells, exhausted T cells, and plasma cells, whereas correlations with NK and TEM cells did not reach statistical significance. Given this positive association with the myeloid compartment, we specifically examined the relationship between Spark-like T cells and M1 macrophages. Scatter plot analysis suggested a strong positive correlation between M1 macrophage module scores and Spark-like T cell proportions at the ROI level (**Figure 6D**), indicating ROI-level spatial co-enrichment of Spark-like T cells with pro-inflammatory myeloid cells. Finally, GO-BP enrichment analysis of regions with high Spark-like T cell intensity identified overrepresentation of sensory perception pathways, including detection of chemical stimulus, olfactory perception, and visual perception, alongside cilium movement, flagellum-dependent cell motility, sperm motility, and regulation of postsynaptic membrane potential (**Figure 6E**). The biological relevance of these enrichment results is currently unclear. Therefore, we interpret them as descriptive findings on the transcriptional composition of Spark-like T cell-enriched regions, and the underlying driving genes and their functional significance warrant further investigation.

**Figure 6:**
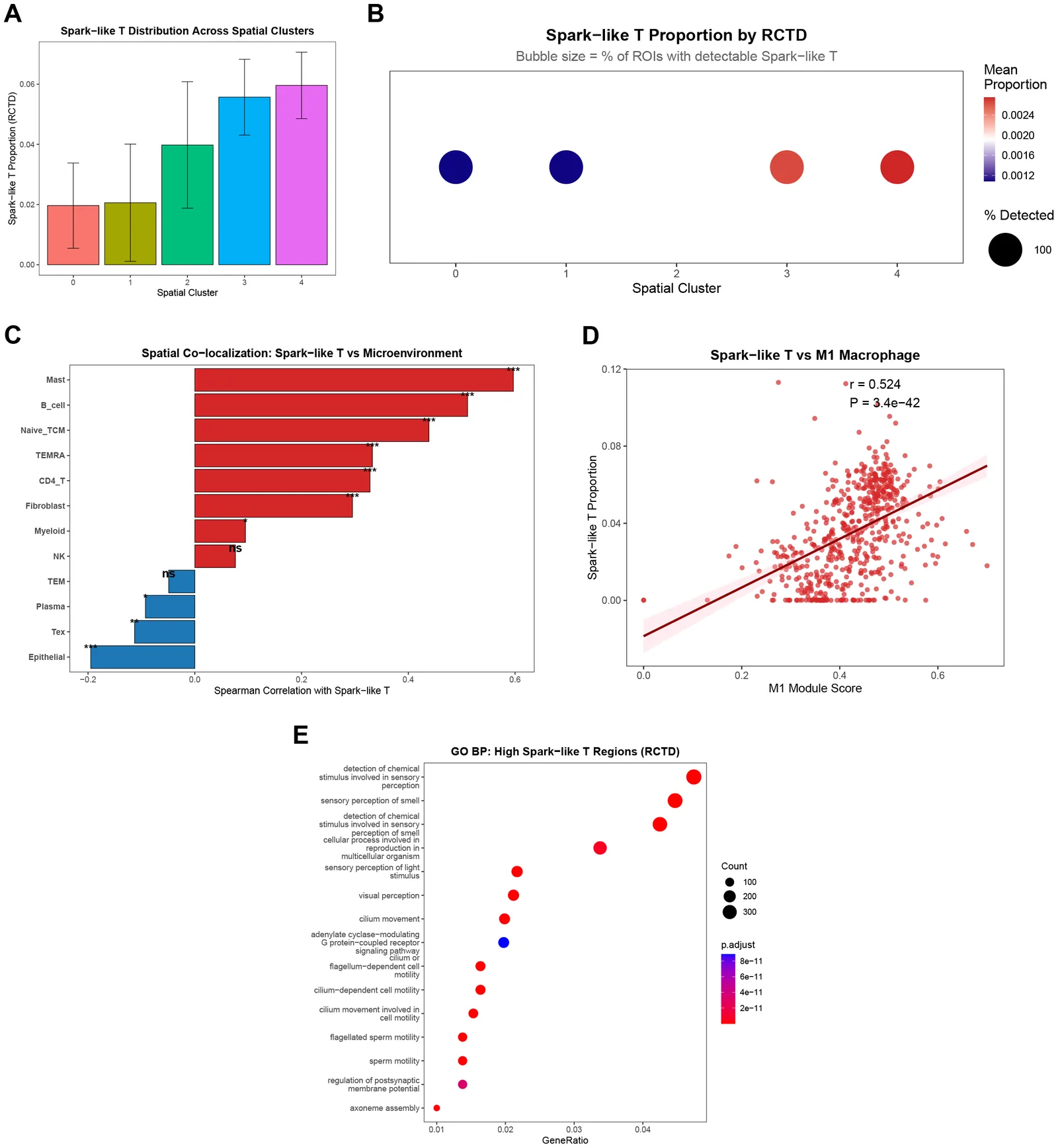
Spatial transcriptomics indicates the spatial distribution of Spark-like T cells and their colocalization characteristics with the immune microenvironment. **(A)** Bar chart showing the distribution of the Spark-like T cell proportion estimated by RCTD deconvolution across different spatial clusters. Different colors represent different spatial clusters. **(B)** Bubble chart showing the proportion of the Spark-like T cell distribution in different spatial clusters. The sizes of the bubbles represent the rate of Spark-like T cell detection in spatial clusters. Red means high distribution rate while dark blue means low distribution rate of Spark-like T cells in each spatial cluster. **(C)** Bar plot showing the spatial co-location between Spark-like T cells and other types of cells in the tumor microenvironment by spearman’s correlation analysis. The correlation coefficients are indicated in the vertical axis, and the names of cell types are indicated in the abscissa axis. Red means positive correlation while blue means negative correlation. **(D)** Scatter plot showing the ROI-level co-localization between Spark-like T cells and M1 macrophages by spearman’s correlation analysis. Each dot represents a spatial location (ROI). The vertical axis indicates M1 macrophages module score, and the abscissa axis indicates the proportion of Spark-like T cells in each spatial location. **(E)** Bubble plot of GO-BP enrichment analysis for spatial regions with high intensity of Spark-like T cells. The names of the biological processes are shown on the abscissa axis, and the gene ratios are indicated on the vertical axis. The sizes of the bubbles mean the number of gene enriched in each biological process. Red color of the dots means higher p-value, while blue means lower p-value. *p< 0.05; **p< 0.01; ***p< 0.001.

### 3.9 Construction and validation of the prognostic signature based on the marker genes of Spark-like T cells

To further investigate the clinical predictive value of Spark-like T cell-related markers, these markers were used to construct a prognostic risk score signature. First, univariate Cox regression analysis was performed in the training cohort TCGA-NSCLC, and 53 genes associated with OS were identified and presented using a forest plot (**Supplementary Figure 10A**). Because including several genes would hinder clinical detection, we used least absolute shrinkage and selection operator (Lasso) regression to reduce the number of genes in the model, and the trajectory of each independent variable was also obtained (**Figure 7A**). As lambda increased, the coefficients of the independent variables gradually decreased to zero (**Figure 6A**). Tenfold cross-validation was applied to build the model, and the confidence intervals for each lambda value are shown in **Figure 7B**. Finally, the optimal model included 14 genes. Therefore, we selected the 14 genes for subsequent analyses and constructed a risk score signature according to the coefficients and expression levels of these genes (**Figure 7C**). The risk score was calculated as follows:

**Figure 7:**
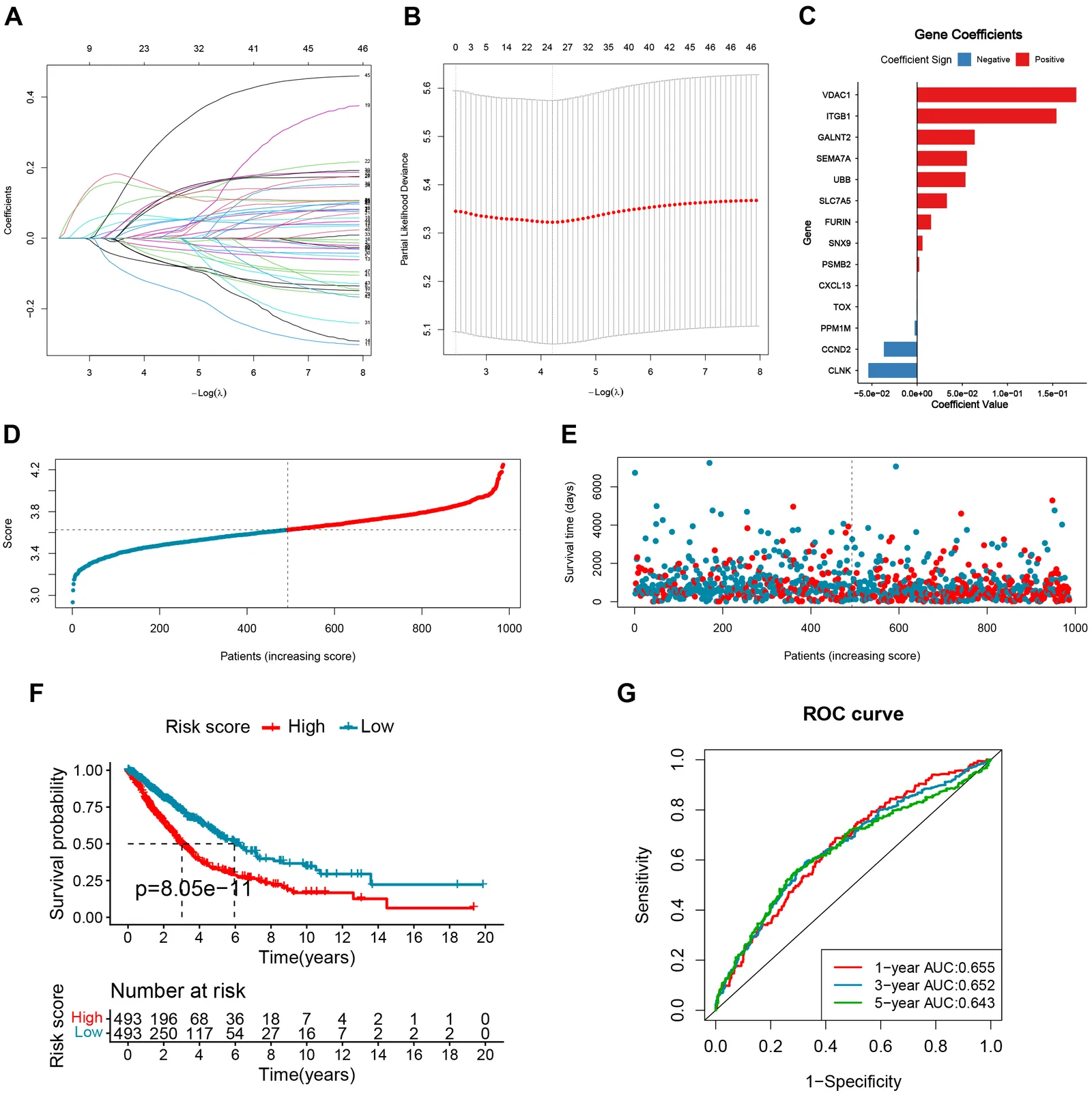
Construction of the Spark-like T cell-related 14-gene prognostic signature in the training cohort. **(A)** Scatter plot showing the trajectory of each independent variable. The abscissa axis represents the -log values of the independent variable lambdas. The vertical axis indicates the coefficients of the independent variables. **(B)** The dynamic process diagram of variables screened by LASSO regression analysis and the selection process diagram of the cross-validation parameter lambda. **(C)** Coefficient of each gene involved in the prognostic model. The abscissa axis represents coefficients, and the vertical axis indicates the gene names. **(D)** The risk score distributions of the patients. Red dots represent patients in high-risk score group while blue dots represent patients in low-risk score group. **(E)** Survival status of the patients. Red dots represent patients who are dead while blue dots represent patients who are still alive. **(F)** The overall survival curve of patients in the high- and low-risk score groups. The abscissa axis shows the survival time, whereas the vertical axis shows the survival probability. The blue curve represents patients in the low-risk score group, whereas the red curve represents patients in the high-risk score group. The grouping status of the patients is indicated at the bottom of the chart. Log-rank test analysis showed the p-value was 8.05e-11. **(G)** ROC curve for predicting the 1-, 3-, and 5-year survival of patients according to the risk scores. The abscissa axis represents specificity, and the vertical axis represents sensitivity. Different colors represent different predictive times.

Risk score = (0.176054579 * VDAC1 expression level) + (0.154084702 * ITGB1 expression level) + (0.063665148 * GALNT2 expression level) + (0.055070655 * SEMA7A expression level) + (0.053453195 * UBB expression level) + (0.032836871 * SLC7A5 expression level) + (0.015427467 * FURIN expression level) + (0.005895955 * SNX9 expression level) + (0.002280775 * PSMB2 expression level) + (-0.000377017 * CXCL13 expression level) + (-0.000389156 * TOX expression level) + (-0.002813987 * PPM1M expression level) + (-0.036721522 * CCND2 expression level) + (-0.053954496 * CLNK expression level).

Using the 14-gene risk score signature, the samples in the TCGA-NSCLC training cohort were divided into two groups, high-risk and low-risk according to the median risk score (**Figure 7D**; **Supplementary Table 7**), and the proportion of patients who had died in the high-risk group was higher (**Figure 7E**). Survival analysis indicated that patients in the high-risk group had significantly shorter OS than patients in the low-risk score group (log-rank test, p = 8.05e-11) (**Figure 7F**). The ROC curve showed that the AUCs for predicting 1-, 3-, and 5-year survival of patients with NSCLC were 0.655, 0.652, and 0.643, respectively (**Figure 7G**), indicating moderate prognostic discrimination.

To test the robustness and generalizability of the 14-gene Spark-like T cell-related prognostic signature constructed in the training cohort, the predictive efficacy of the signature was validated in several independent external datasets using the same algorithm. The results showed that patients in the high-risk score group had significantly shorter OS than those in the low-risk score group, and the risk score model showed moderate prognostic discrimination, with time-dependent AUCs of approximately 0.57-0.69 across the validation cohorts (**Figures 8A–F**): The AUCs for 1-, 3-, and 5- year survival were 0.572, 0.601, and 0.618, respectively, in validation cohort GSE37745 (**Figure 8B**); the AUCs for 1-, 3-, and 5- year survival were 0.681, 0.628, and 0.615, respectively, in validation cohort GSE41271 (**Figure 8D**); the AUCs for 1-, 3-, and 5- year survival were 0.617, 0.674, and 0.691, respectively, in validation cohort GSE72094 (**Figure 8F**). To examine whether the risk score model was an independent prognostic factor for GC patients, multivariate Cox regression analyses using the “coxph()” function in the R package “survival” were performed. Across the training and validation cohorts, the risk score was an independent prognostic factor after accounting for clinical features, such as age, sex and tumor stage (**Figures 9A–D**). These results indicated that the 14-gene prognostic signature based on the Spark-like T cell-related markers was robust and generalizable in predicting the prognosis of patients with NSCLC.

**Figure 8:**
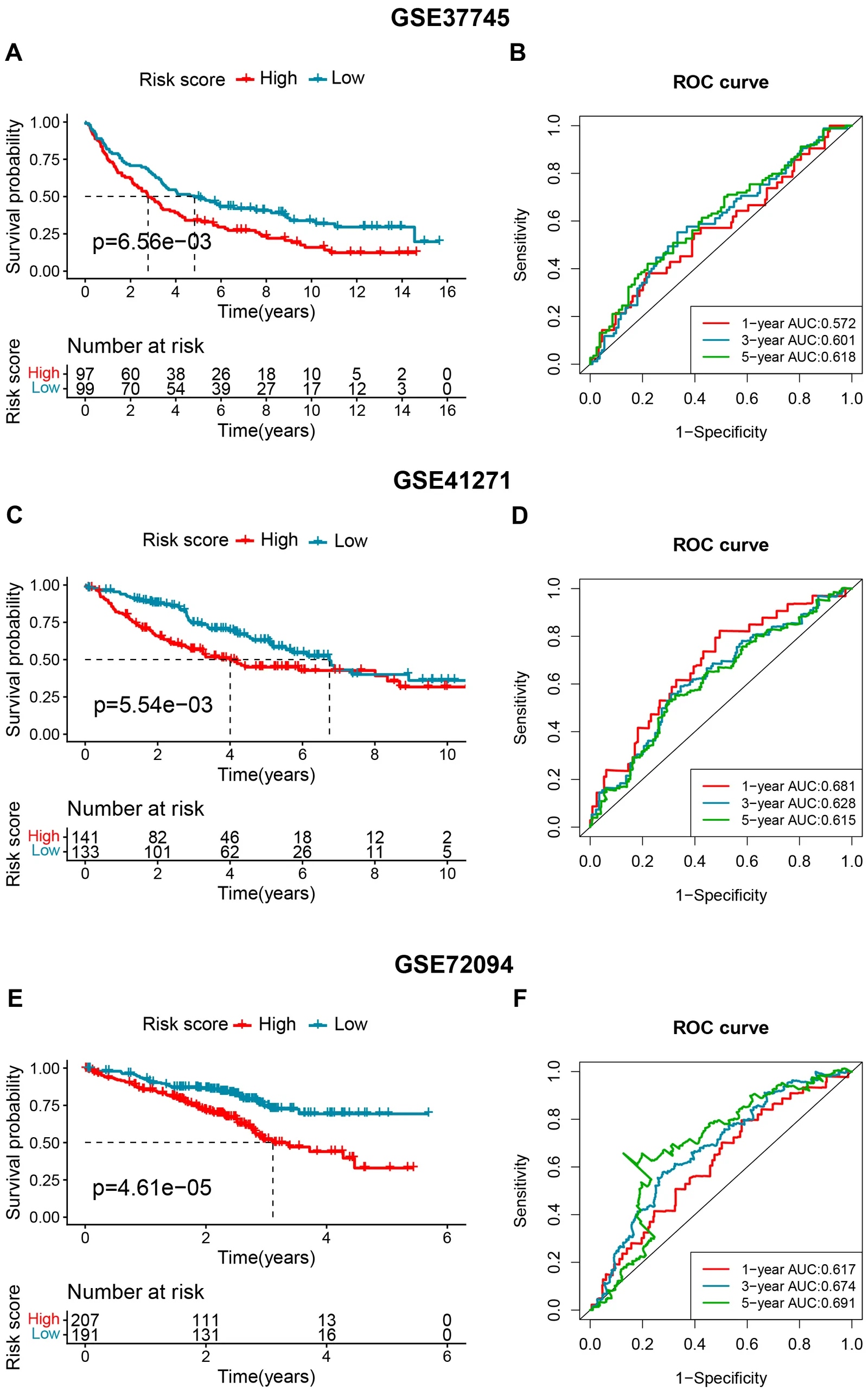
Validation of the predictive efficacy of the 14-gene prognostic model in external independent cohorts. **(A)** The OS curve of patients in the high- and low-risk score groups in GSE37745 cohort. **(B)** The ROC curve for predicting the 1-, 3-, and 5-year survival of patients according to the risk score in GSE37745 cohort. **(C)** The OS curve of patients in the high- and low-risk score groups in GSE41271 cohort. **(D)** The ROC curve for predicting the 1-, 3-, and 5-year survival of patients according to the risk score in GSE41271 cohort. **(E)** The OS curve of patients in the high- and low-risk score groups in GSE72094 cohort. **(F)** The ROC curve for predicting the 1-, 3-, and 5-year survival of patients according to the risk score in GSE72094 cohort. For the OS curves in **(A, C, E)**, the abscissa axis shows the survival time, whereas the ordinate axis shows the survival probability. The blue curve represents patients in low-risk score group, whereas the red curve represents patients in high-risk score group. The grouping status of the patients is indicated at the bottom of the chart. For the ROC curves in **(B, D, F)**, The abscissa axis represents specificity, and the vertical axis represents sensitivity. Different colors represent different predictive times. OS, overall survival; ROC, receiver operating characteristic curve.

**Figure 9:**
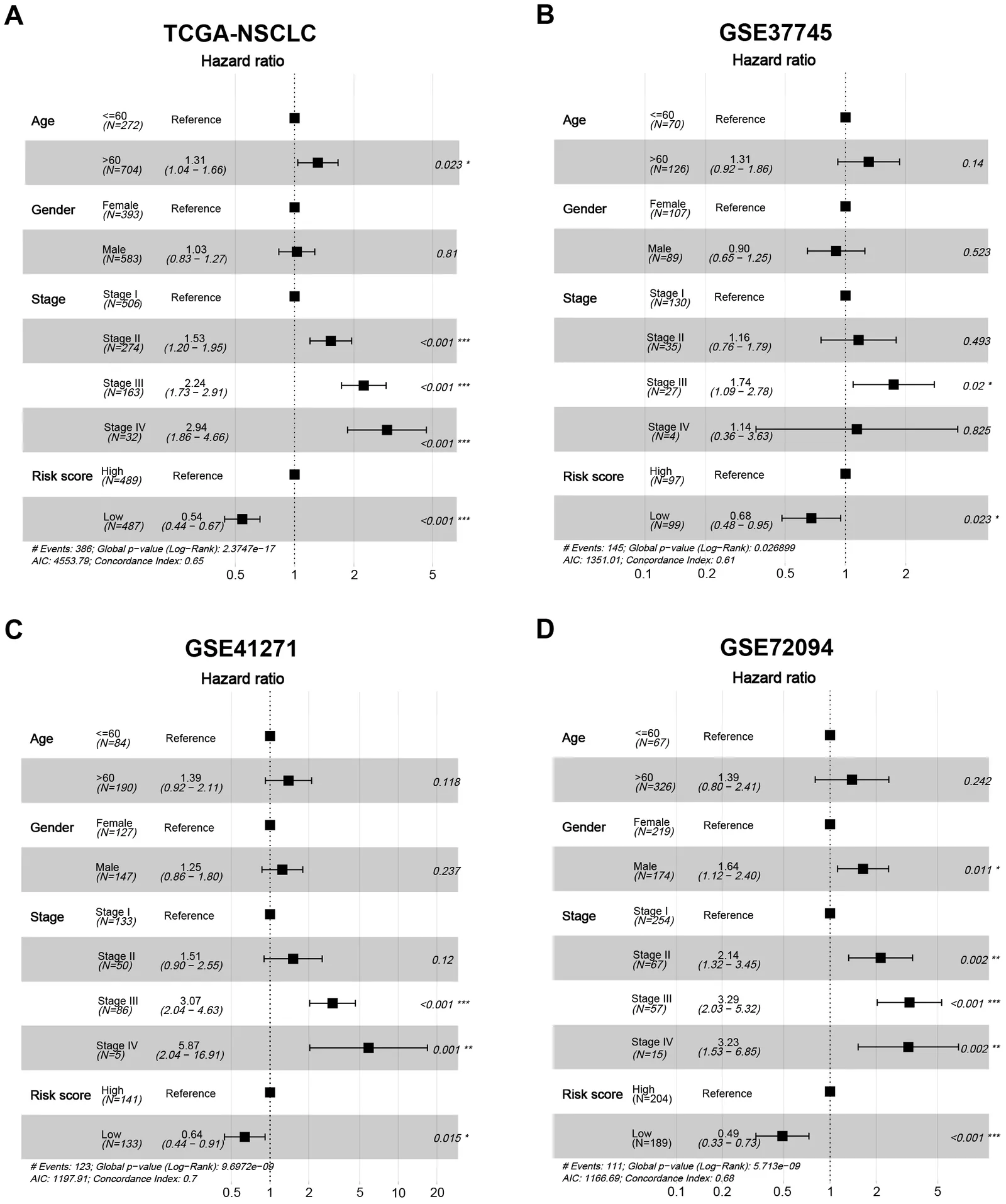
Forest plots showing that the risk score was an independent prognostic factor through multivariate Cox regression analysis in the training cohort **(A)** TCGA-NSCLC, and the validation cohort: **(B)** GSE37745, **(C)** GSE41271, **(D)** GSE72094. The names of the variables are on the left of each plot. The hazard ratio of each variable and the corresponding forest plot are in the middle of each plot. The p-values of the corresponding variables are on the right of each plot.

### 3.10 Exploratory analysis of biological features associated with the Spark-like T cell-related prognostic signature

To explore potential biological processes associated with the relationships between the Spark-like T cell-related signature and the prognosis of patients with NSCLC, functional enrichment analyses were performed using GSVA. The results suggested that KEGG pathways associated with cancer development were more active in the high-risk score group, such as the TGF-β signaling pathway, pathways in cancer and the ERBB signaling pathway (**Figure 10A**; **Supplementary Table 8**). All the oncogenic signature gene sets and gene sets from the Curated Cancer Cell Atlas exhibited significantly higher activity in the high-risk score group (**Figures 10B, C**; **Supplementary Table 8**). We next compared ESTIMATE-derived tumor microenvironment scores between the high- and low-risk groups. The immune score reflects inferred immune cell infiltration, whereas the stromal score reflects inferred stromal content. The results showed that patients with low risk scores tended to have higher immune scores, although the difference did not reach statistical significance (P = 0.077; **Figure 9D**), and lower stromal scores (**Figure 9E**). To further explore the correlation between the risk score and tumor immune characteristics, we quantified the infiltration of different immune cell types in bulk samples using the CIBERSORT algorithm. The results showed that the infiltration of anti-tumoral immune cells, such as CD8 T cells, naive B cells, and resting dendritic cells was higher in the low-risk score group compared with the high-risk score group (**Figure 10F**, **Supplementary Table 9**). These findings suggested that the TME landscape differed between patients with NSCLC in the high- and low-risk score groups, which may contribute to the heterogeneity in clinical outcomes.

**Figure 10:**
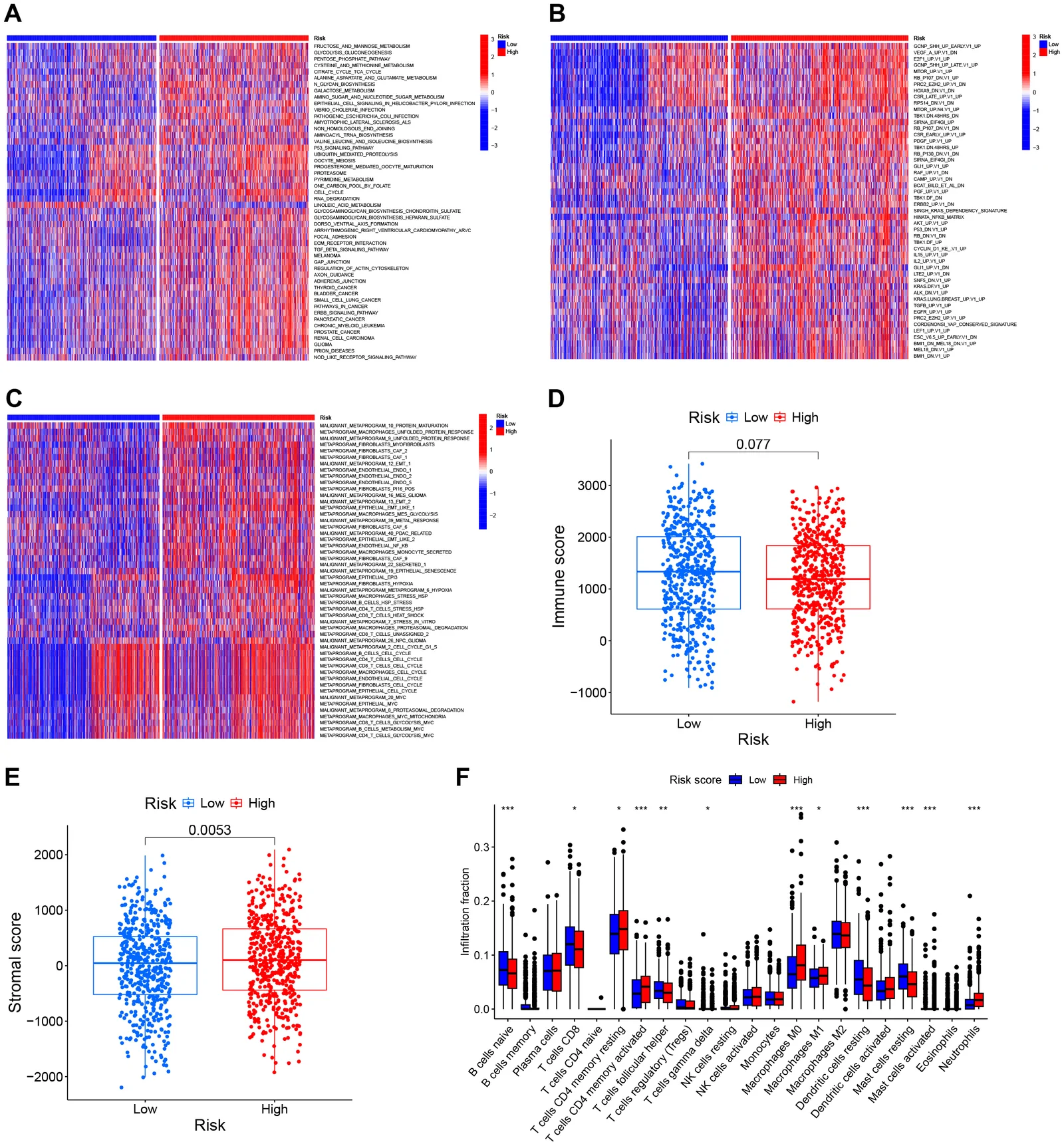
Association between the risk score and the characteristics of tumor microenvironment. **(A)** The GSVA results of KEGG pathway gene sets indicated the variation of biological pathways activation between the low- and high-risk score groups. **(B)** The GSVA results indicated differentially activated oncogenic signature gene sets between the low- and high-risk score groups. **(C)** The GSVA results of gene sets from the Curated Cancer Cell Atlas indicated differentially activated hallmarks of cancer between the low- and high-risk score groups. In **(A–C)**, The risk score groups of patients are annotated at the top of the panel. The biological processes and pathways are listed on the right. Red represents activation, whereas blue represents inhibition. **(D, E)** Box plots showing the differences of **(D)** immune score and **(E)** stromal score between NSCLC patients in low- and high-risk score groups. Each dot represents a patient. Red represents high-risk score group while blue represents low-risk score group. The grouping status is indicated in the vertical axis, and the relative scores are indicated in the abscissa axis. **(F)** Relative abundances of the 22 types of immune cells in the low-risk score and high-risk score groups. The abscissa axis represents the names of the immune cells. The vertical axis represents the infiltration fraction.

## 4 Discussion

In this study, we performed an integrated multi-omics analysis to characterize CD8^+^T cell heterogeneity associated with anti-PD-1 immunotherapy response in NSCLC. By combining scRNA-seq, mIHC validation, bulk RNA-seq, spatial transcriptomics, trajectory inference, cell-cell communication analysis, and in silico perturbation modeling, we identified a responder-enriched CD8^+^T cell state termed Spark-like T cells. This population was characterized by high expression of CD2, ENTPD1 (CD39), and PTPRC (CD45RO) and low expression of CCR7 and IL7R, together with transcriptional programs related to T cell activation, antigen experience, cytotoxicity, cytokine signaling, and immune regulation. Importantly, Spark-like T cells were enriched in responders to anti-PD-1 therapy at the single-cell level, validated by mIHC in an independent clinical cohort, and further supported by functional enrichment, pseudotime, intercellular communication, spatial localization, and prognostic signature analyses. Collectively, these findings suggest that Spark-like T cells may represent a clinically relevant antitumor CD8^+^T cell state associated with effective immune checkpoint blockade in NSCLC.

A central observation of this study is the preferential enrichment of Spark-like T cells in patients who responded to anti-PD-1 immunotherapy. In the single-cell atlas, UMAP-based visualization and compositional comparison suggested that Spark-like T cells were more abundant in responders, whereas non-responders showed relative predominance of Naive-TCM and Tex populations (**Figures 2B, E, F**). This finding is consistent with previous studies showing that the efficacy of PD-1/PD-L1 blockade depends on both the total number of tumor-infiltrating lymphocytes and the functional state, clonal expansion, and spatial organization of tumor-reactive CD8^+^T cells (7–10, 12, 13). In NSCLC and other solid tumors, pre-existing activated or reinvigorated cytotoxic T cell populations are often associated with better therapeutic outcomes, whereas dysfunctional or terminally exhausted states may limit durable responses (7, 9, 13–15). Our results extend these observations by defining a specific CD8^+^T cell program enriched in responders, beyond broad immune-cell categories.

The mIHC validation further supports the clinical relevance of this Spark-like T cell state. Using a CD8A^+^CD39^+^CD45RO^+^ surrogate immunophenotype consistent with the transcriptomically defined Spark-like T cell state, we confirmed that tumor tissues from patients with a partial response exhibited greater Spark-like T cell infiltration than those from patients with stable disease (**Figures 3A, B, J**). Moreover, Kaplan-Meier analyses showed that Spark-like T-cell intensity was associated with patient survival, comparable to established clinicopathological parameters such as T stage, N stage, and clinical stage (**Figures 3C–F**). The relationship between Spark-like T cell infiltration and tumor stage was also evaluated by quantitative mIHC analysis (**Figures 3G–I**), suggesting that this population may provide clinically meaningful information beyond conventional staging. CD39 has been reported to identify antigen-experienced, tumor-reactive CD8^+^T cells in human tumors, while CD45RO marks memory T cell differentiation (16, 17). Therefore, the CD8A^+^CD39^+^CD45RO^+^ phenotype used in our validation cohort is biologically compatible with a tumor-experienced effector/memory-like T-cell state. Importantly, stage-stratified survival analyses demonstrated that, among patients with the same T stage, N stage, or clinical stage, those with high tumoral infiltration of Spark-like T cells still tended to have a better prognosis (**Figures 4A–I**), and multivariable Cox regression further confirmed that high Spark-like T cell infiltration was an independent protective factor for OS after adjustment for tumor stage and other clinical variables (**Figure 4J**). These results suggested that Spark-like T cells may serve as an independent tissue-based biomarker for immunotherapy responsiveness in NSCLC.

Functional annotation of Spark-like T cells indicated that this population is linked to immune activation and effector function. Marker gene analyses showed that Spark-like T cells had a transcriptional profile distinct from those of other CD8^+^T cell populations (**Supplementary Figure 5A**; **Supplementary Table 3**). GO-BP and KEGG enrichment analyses further indicated that Spark-like marker genes were involved in immune-related biological processes and signaling pathways (**Supplementary Figures 5B, C**). In parallel, differential expression analysis between responders and non-responders identified immune-response-associated transcriptional remodeling in CD8^+^T cells (**Supplementary Figure 5D**; **Supplementary Table 1**). The enrichment analyses shown in **Supplementary Figures 3E–H** further suggested that responder-associated CD8^+^T cell programs were associated with pathways, such as T cell activation, immune effector function, antigen processing/presentation, cytokine signaling, and cytotoxic immune responses. These findings are consistent with the established mechanism of PD-1 blockade, which can restore antitumor T cell activity and enhance effector cytokine production and cytotoxic killing (6, 10, 12, 13).

The pseudotime analysis provided additional insight into the developmental position of Spark-like T cells within the CD8^+^T cell continuum. Trajectory reconstruction indicated dynamic differentiation patterns among tumor-infiltrating CD8^+^T cells (**Supplementary Figure 6A**). Cells from responders and non-responders showed different distributions along the inferred trajectory (**Supplementary Figures 6B, C**), and pseudotime-dependent genes displayed distinct expression dynamics between the two groups (**Supplementary Figure 6D**). Notably, key markers including CCR7, IL7R, ENTPD1, CD2, and PTPRC varied with pseudotime in a subtype-dependent manner (**Supplementary Figure 6E**). Comparison of pseudotime values further suggested that Spark-like T cells from responders and non-responders may occupy different functional or developmental states (**Supplementary Figure 6F**). These observations are compatible with current models of CD8^+^T cell differentiation in tumors, in which naïve/central-memory-like, progenitor exhausted, effector-like, and terminally exhausted states coexist and may respond differently to checkpoint blockade (7, 9, 14, 15, 18). Spark-like T cells may therefore represent an intermediate or activated antigen-experienced state with enhanced responsiveness to PD-1 inhibition. Within the framework of the CD8^+^T cell exhaustion continuum spanning from progenitor to terminally differentiated exhausted states (11), our data place Spark-like T cells closer to an antigen-experienced transitional state than to terminally exhausted cells. Nevertheless, owing to the limited sample size of the scRNA-seq cohort and incomplete marker coverage, we could not reliably subdivide the exhausted compartment into progenitor-, intermediate-, and terminally exhausted populations for a direct quantitative comparison; this remains an important question for future studies with larger, higher-resolution datasets.

Cell-cell communication analysis indicated that Spark-like T cells may participate in a broader immunoregulatory network within the tumor microenvironment. Global interaction numbers and strengths differed between responders and non-responders (**Supplementary Figure 7A**), as well as differential Spark-like T cell-related interaction patterns with other cell populations (**Supplementary Figure 7B**). Further pathway-level analysis suggested that in responders, Spark-like T cells and other immune cells exhibited prominent outgoing signaling through pathways such as MHC-I, MHC-II, CCL, CD99, GALECTIN, PARs, CD45, and LCK, while receiving incoming signals through MHC-I, MHC-II, GALECTIN, CD45, FN1, and IL16 pathways (**Supplementary Figures 8A, B**). The corresponding network plots illustrated complex communication relationships among immune-cell subtypes (**Supplementary Figures 8C, D**). These findings are biologically plausible, as effective antitumor immunity requires coordinated interactions among CD8^+^T cells, antigen-presenting cells, macrophages, stromal cells, and tumor cells (19–21). In particular, enrichment of MHC-related signaling suggests enhanced antigen-recognition capacity, whereas CCL and IL16 pathways may contribute to immune-cell recruitment and spatial immune organization. Thus, Spark-like T cells may not act in isolation but instead may represent part of a responder-associated immune ecosystem. It should be noted that both the scRNA-seq and mIHC tumor samples were collected from surgical specimens after neoadjuvant anti-PD-1-based therapy. Therefore, the enrichment of Spark-like T cells in responders may therefore represent a treatment-associated state rather than a baseline predictive biomarker, and its value as a pre-treatment predictor remains to be established.

The spatial transcriptomic analysis further strengthens this interpretation. Although scRNA-seq provides high-resolution cellular phenotyping, spatial context is lost during tissue dissociation. By integrating spatial transcriptomics, we mapped the tissue distribution and microenvironmental co-localization of Spark-like T cell-related signals. This spatial context matters because the clinical effect of CD8^+^T cells depends strongly on their localization relative to tumor nests, stromal barriers, antigen-presenting cells, and suppressive myeloid compartments (22–24). Prior studies have shown that inflamed, immune-excluded, and immune-desert tumors differ markedly in response to immune checkpoint blockade (22, 23). Our spatial findings indicate that Spark-like T cells preferentially co-localize with immune and stromal compartments at the ROI level. Whether this represents a specific ecological niche that actively sustains the Spark-like T cell state, or instead reflects their accumulation within generally inflamed, immune- and stroma-rich regions, cannot be resolved at the present resolution and requires further functional and higher-resolution spatial analyses.

To move beyond descriptive characterization, we performed multidimensional screening of candidate regulatory targets in Spark-like T cells. By integrating five evidence layers, including differential expression, pseudotime trajectory, ligand-receptor interactions, pathway involvement, and prognosis, we prioritized genes potentially central to the Spark-like program (**Figure 5A**; **Supplementary Table 5**). Among the top candidates, TNF, ENTPD1, IL2, IFNG, GZMB, and RUNX3 are particularly relevant to T-cell biology. IFNG and GZMB are canonical mediators of cytotoxic antitumor activity, IL2 supports T-cell proliferation and survival, TNF contributes to inflammatory and cytotoxic responses, RUNX3 is a key transcriptional regulator of cytotoxic CD8^+^T cell differentiation, and ENTPD1 (CD39) marks antigen-experienced tumor-infiltrating T cells while also participating in purinergic immune regulation (16, 25–27). These genes may therefore represent both biomarkers and functional components of the Spark-like T cell state.

The in silico KO analysis provided hypothesis-generating evidence for the functional necessity of these core genes. Using a scTenifoldKnk-style virtual KO strategy, we assessed the impact of single-gene and combined multi-gene perturbations on the Spark Core module score (**Supplementary Table 6**). Both individual gene perturbation and combined KO of multiple core genes, altered the Spark-like module, suggesting that this transcriptional state is maintained by a coordinated regulatory network rather than by a single marker. This is consistent with systems-level models of T cell activation and exhaustion, in which transcription factors, cytokines, checkpoint molecules, metabolic enzymes, and effector genes interact to determine functional phenotypes (6, 7, 9, 10, 12–15). Although computational KO cannot replace experimental perturbation, it provides a rational framework for prioritizing genes for future validation using CRISPR screening, T cell co-culture systems, organoid models, or *in vivo* immunotherapy models.

Another translational contribution of this study is the construction of a 14-gene Spark-like T cell-related prognostic signature (**Figures 7A–G**; **Supplementary Figure 10A**; **Supplementary Table 7**). Among the 14 genes included in the signature, VDAC1, ITGB1, GALNT2, SEMA7A, UBB, SLC7A5, FURIN, SNX9, and PSMB2 were adverse prognostic factors in patients with NSCLC, while CXCL13, TOX, PPM1M, CCND2, and CLNK were protective prognostic factors in NSCLC (**Figure 7C**; **Supplementary Figure 10A**). Previous reports indicate that VDAC1, ITGB1, GALNT2, SEMA7A, UBB, SLC7A5, FURIN, and SNX9 are oncogenes and contribute to NSCLC progression and poor prognosis (28–35), whilst CXCL13, TOX, CCND2, and CLNK could suppress the progression of NSCLC through various mechanisms (36–39). These reports are consistent with our findings (**Figure 7C**; **Supplementary Figure 10A**). However, the functions of PSMB2 and PPM1M remain underexplored and warrant further investigation. Using the prognostic signature, patients in the training cohort were divided equally into high-risk and low-risk score groups (**Figures 7D, E**; **Supplementary Table 7**), with the high-risk group showing significantly shorter overall survival (OS) than the low-risk group (**Figure 7F**). The model showed moderate prognostic discrimination, with time-dependent AUCs of approximately 0.57–0.69, which were validated in multiple external datasets (**Figure 7G**, **Figures 8A–F**). The signature was an independent prognostic factor after adjustment for age, sex, and tumor stage (**Figures 9A–D**). Functional enrichment showed that high-risk tumors were enriched for TGF-β, ERBB, and oncogenic signatures (**Figures 10A–C**), whereas low-risk tumors tended to have higher immune scores, albeit without reaching statistical significance (P = 0.077; **Figure 9D**), and showed greater infiltration of anti-tumoral immune cells such as CD8^+^T cells, naive B cells, and resting dendritic cells (**Figures 10D–F**; **Supplementary Tables 8**, **9**). Collectively, these findings indicate that the Spark-like T cell-related prognostic signature provides robust and effective prognostic prediction for patients with NSCLC, and link the Spark-like T cell program to the broader immune landscape, suggesting that the signature captures clinically meaningful TME heterogeneity.

These findings have potential therapeutic implications. If Spark-like T cells represent an immune-active, responder-associated CD8^+^T cell state, then therapeutic strategies that induce, expand, or preserve this population may improve the efficacy of PD-1 blockade. Possible approaches include enhancing antigen presentation, improving dendritic cell priming, modulating chemokine-mediated T-cell recruitment, targeting suppressive myeloid or stromal barriers, and combining PD-1 blockade with cytokine or co-stimulation based therapies (19, 20, 40). However, several molecules associated with Spark-like T cells may have context-dependent roles. For example, IFNG and TNF can mediate antitumor immunity but may also drive adaptive resistance mechanisms, including upregulation of immune checkpoints or antigen-presentation alterations (41). Similarly, CD39 identifies tumor-reactive T cells but is also involved in adenosine generation, which can suppress antitumor immunity (16, 42). Therefore, therapeutic manipulation of Spark-like T-cell programs should be guided by careful mechanistic and spatial validation.

Several limitations should be acknowledged. First, the single-cell analysis relied mainly on GSE207422, which has a limited sample size and may not fully capture NSCLC heterogeneity. Second, the mIHC validation cohort was retrospective and recruited at a single center, necessitating prospective multicenter validation. Third, the mIHC quantification relied on a surrogate immunophenotype (CD8A^+^CD39^+^CD45RO^+^). Because CD39 and CD45RO are broad markers of tumor-reactive/antigen-experienced and memory CD8^+^T cells, respectively (15, 16), this surrogate may not fully recapitulate the transcriptomically defined Spark-like T cell state, and additional discriminating markers or functional validation are needed. Fourth, Spark-like T cells were defined computationally using marker-based gating and module scores, and protein-level or functional validation is still lacking. Fifth, owing to the limited sample size and marker coverage of the scRNA-seq dataset, we could not reliably subdivide the exhausted CD8^+^T cell compartment into progenitor-, intermediate-, and terminally exhausted populations, and the relationship between Spark-like T cells and these exhaustion states could therefore not be directly compared. Sixth, the in silico KO results are hypothesis-generating and need experimental confirmation. Finally, the spatial analysis was ROI-level GeoMx DSP data, limiting the resolution of local cell-cell neighborhoods, and the observed co-localization of Spark-like T cells with M1 macrophages therefore represents an ROI-level spatial association rather than a demonstrated functional interaction. Because our study was mainly based on transcriptomic, spatial and immunohistochemical analyses, it could identify associations but not prove causal mechanisms. Functional experiments are needed to clarify the mechanisms underlying the relationship between Spark-like CD8^+^T cells and NSCLC progression or treatment response.

## 5 Conclusion

This study identifies Spark-like T cells as a clinically relevant CD8+T cell functional state associated with a favorable response to anti-PD-1 immunotherapy and improved survival in NSCLC. Detection of this population may help refine patient stratification, complement existing biomarkers, and inform the assessment of immunotherapy benefit. Moreover, the Spark-like T cell-related signature provides a potential translational tool for risk assessment and clinical decision-making.

## Data Availability

The RNA-seq data and relative clinical information of the TCGA-LUAD and TCGA-LUSC cohorts were downloaded from the TCGA database (https://portal.gdc.cancer.gov/). The GEO datasets were downloaded from the GEO database (https://www.ncbi.nlm.nih.gov/gds/) under the accession number(s) GSE37745, GSE41271, GSE72094, GSE207422, and GSE271689.
